# *Sasa quelpaertensis* Leaf Extract Ameliorates Dyslipidemia, Insulin Resistance, and Hepatic Lipid Accumulation in High-Fructose-Diet-Fed Rats

**DOI:** 10.3390/nu12123762

**Published:** 2020-12-07

**Authors:** Jeong Yong Park, Mi Gyeong Jang, Jung Min Oh, Hee Chul Ko, Sung-Pyo Hur, Jae-Won Kim, Songyee Baek, Se-Jae Kim

**Affiliations:** 1 Department of Biology, Jeju National University, Jeju 63243, Korea; jjjoosd@naver.com (J.Y.P.); mkjang@jejunu.ac.kr (M.G.J.); ojh554@naver.com (J.M.O.); 2 Biotech Regional Innovation Center, Jeju Nation University, Jeju 63423, Korea; ifly1007@jejunu.ac.kr (H.C.K.); kjw8839@jejunu.ac.kr (J.-W.K.); summerbee@jejunu.ac.kr (S.B.); 3 Jeju International Marine Science Research & Logistics Center, Korea Institute of Ocean Science & Technology, Gujwa, Jeju 63349, Korea; hursp@kiost.ac.kr

**Keywords:** *Sasa quelpaertensis*, dyslipidemia, high-fructose diet, insulin resistance, metabolic dysfunction

## Abstract

Background: Increased dietary fructose consumption is closely associated with lipid and glucose metabolic disorders. *Sasa quelpaertensis* Nakai possesses various health-promoting properties, but there has been no research on its protective effect against fructose-induced metabolic dysfunction. In this study, we investigated the effects of *S. quelpaertensis* leaf extract (SQE) on metabolic dysfunction in high-fructose-diet-fed rats. Methods: Animals were fed a 46% carbohydrate diet, a 60% high-fructose diet, or a 60% high-fructose diet with SQE (500 mg/kg of body weight (BW)/day) in drinking water for 16 weeks. Serum biochemical parameters were measured and the effects of SQE on hepatic histology, protein expression, and transcriptome profiles were investigated. Results: SQE improved dyslipidemia and insulin resistance induced in high-fructose-diet-fed rats. SQE ameliorated the lipid accumulation and inflammatory response in liver tissues by modulating the expressions of key proteins related to lipid metabolism and antioxidant response. SQE significantly enriched the genes related to the metabolic pathway, namely, the tumor necrosis factor (TNF) signaling pathway and the PI3K-Akt signaling pathway. Conclusions: SQE could effectively prevent dyslipidemia, insulin resistance, and hepatic lipid accumulation by regulation of metabolism-related gene expressions, suggesting its role as a functional ingredient to prevent lifestyle-related metabolic disorders.

## 1. Introduction

High-energy food intake along with a sedentary lifestyle is known to be a major cause of the current obesity epidemic, metabolic syndrome, nonalcoholic fatty liver disease (NAFLD), and type 2 diabetes [1,2,3,4]. In particular, dietary fructose consumption over the last century has been associated with the increased prevalence of obesity, insulin resistance, and NAFLD, a liver manifestation of metabolic syndrome [5]. Several studies have indicated that the consumption of fructose is associated with increased visceral adiposity, insulin resistance, hepatic de novo lipogenesis (DNL), and hepatic inflammation [6,7,8,9]. 

Fructose is a particularly lipogenic sugar due to its characteristic metabolism in humans. Since fructose is absorbed via the portal vein, the liver contains it in much higher concentrations compared with other tissues. Within the hepatocytes, fructose is rapidly phosphorylated by fructokinase, which is not inhibited by ATP and might not be as responsive to cellular energy states [4,10]. In fructolysis, the carbons of fructose become triglycerides by activating DNL enzymes. Fructose promotes the production of reactive oxygen species (ROS) through ATP depletion and the suppression of mitochondrial fatty acid oxidation [3,11]. Due to the high prevalence of metabolic syndrome and the lack of satisfactory treatments for it, there is increasing interest in alternative medicine and natural herbs that produce many beneficial and healing properties without any adverse effects.

The genus *Sasa* (Poaceae) is composed of perennial plants commonly known as dwarf bamboo; various *Sasa* spp. are distributed throughout Asian countries including China, Japan, Korea, and Russia [12]. Their leaves have been used in traditional medicine for the treatment of gastric ulcers, dipsosis, and hematemesis due to their anti-inflammatory, antipyretic, and diuretic properties [13]. The leaves of several *Sasa* species, such as *S. kurilensis, S. senanensis, S. borealis*, are reported to possess various health-promoting properties, including anticancer and antioxidant effects [14,15,16,17,18]. *S. quelpaertensis* Nakai is a species of dwarf bamboo that grows on Mount Halla on Jeju Island, Republic of Korea. Its leaf extract has antiobesity, antidepressant, antivirus, and anticancer properties [19,20,21,22]. Kim et al. [23] reported that its leaf extract improved the lipid profiles by modulating lipid metabolism in high-fat-diet-fed rats. However, few studies have investigated the protective effect of *S. quelpaertensis* leaf extract (SQE) against high-fructose-diet-induced metabolic disorders. In this study, we investigated whether SQE could prevent insulin resistance, dyslipidemia, and hepatic lipid accumulation in high-fructose-diet-fed rats. 

## 2. Materials and Methods 

### 2.1. Plant Material and Preparation of SQE

*Sasa quelpaertensis* leaves were collected from Mt. Halla on Jeju Island, Republic of Korea in September 2019. *S. quelpaertensis* extract (SQE) was prepared according to Lee et al. [24]. The leaves were washed, dried at 60 °C for 24 h, and pulverized into powder of 200 mesh. The resulting powder was extracted at 90 °C for 4 h with water and the soluble extract was removed. The remaining residue was dried and extracted with 70% ethanol. SQE was concentrated, freeze-dried, and stored at –70 °C until its use. The composition of SQE was analyzed by an analytical HPLC instrument (Water 2695 Alliance system, Milford, MA, USA) equipped with a photodiode array detector at 320 nm. SQE (20 mg/mL) was analyzed using XBridge BEH C_18_ column (4.6 × 250 mm, 5 µm). Chromatographic separation was performed using gradient elution at a flow rate of 0.8 mL/min with mobile phase for 0.5% acetic acid in distilled water (A)/0.5% acetic acid in acetonitrile (B) as follows: 0.0 min, 85.0% A and 15.0% B; 40.0 min, 57.5% A and 42.5% B; 40.1–45.0 min, 100% B; 45.0–55 min, 85.5% A. The separation was carried out at 40 °C with a sample injection volume of 10 µL and a flow rate of 0.8 mL/min. The SQE contained various phytochemicals, including tricin (7.43 mg/g) and *p*-coumaric acid (2.69 mg/g), which were the major components [20] and were used as indicator components (Figure S1).

### 2.2. Animals and Diet

Four-week-old male Sprague–Dawley (SD) rats were obtained from Orient Bio Inc. (Seongnam, Korea). The animals were acclimated for 3 weeks by housing them in a room maintained at 23 ± 1 °C with relative humidity of 60% ± 5% and a light/dark cycle of 12 h. Animal experiments were approved by the Jeju National University Institutional Animal Care and Use Committee (Approval number: 2019-0008) and carried out in accordance with the guidelines for animal care of the Institute. At the end of the acclimation, the rats were randomly divided into three groups (*n* = 7/group) and fed a high-carbohydrate diet (HC group), or a high-fructose diet without (HF group) or with SQE (SQE group) for 16 weeks. The composition of the experimental diet is shown in Table S1. For the SQE group, SQE was administered to high-fructose-fed rats at a dose of 500 mg/kg of BW between 10:00 and 11:00 a.m. once daily through drinking water for 16 weeks. 

### 2.3. Biochemical Parameter Assays

On the final experimental day, all rats were euthanized with carbon dioxide. Blood samples were drawn from the heart into a syringe and allowed to stand at room temperature for 30 min. Serum samples were than collected by centrifugation at 3000× *g* for 10 min. The tissues were extracted, snap frozen in liquid nitrogen, and stored at –70 °C. The levels of serum triglyceride, total cholesterol, and LDL/HDL cholesterol were measured by commercial kits (Do gen bio, Seoul, Korea), and the levels of serum GOT/GPT were measured using kits from Asan pharm (Gyeong gi, Korea) according to the manufacturer’s protocols. Fasting serum insulin levels were measured using the Mercodia Rat Insulin ELISA Kit (Mercodia, Uppsala, Sweden). The oral glucose tolerance test (OGTT) was performed on the day after 16 weeks. The experimental animals were fasted for more than 12 h and then glucose (2 g/kg of BW) was administered orally. The blood was collected from the tail vein every 0, 30, 60, 90, and 120 min after oral glucose supplementation, and the glucose levels were measured with a blood glucose tester (Osang Healthcare, Gyeonggi, Korea). The area under the glucose curve (AUC) was calculated by the trapezoidal rule. Insulin resistance was assessed by calculating the HOMA-IR (homeostasis model assessment of insulin resistance) index using the following formula [25]: HOMA-IR = [fasting glucose (mg/dL) × fasting insulin (μg/L)]/22.5.

### 2.4. Western Blot Analysis 

Pieces from the liver were homogenized in cold lysis buffer (1 × RIPA buffer, 1 mM phenylmethylsulfonyl fluoride, 1 mM Na3Vo4, 1 mM NaF, 1 μg/mL aprotinin, 1 μg/mL pepstatin, and 1 μg/mL leupeptin) and collected by centrifugation at 13,000 rpm for 20 min at 4 °C. Lysate protein concentrations were determined using a protein assay featuring a dye reagent (Bio-Rad; Hercules, CA, USA). The proteins were subjected to electrophoresis on 8–10% (*w*/*v*) SDS-polyacrylamide gels and transferred to a polyvinylidene fluoride membrane. The membrane was blocked with 5% bovine serum albumin (Bovogen, Keilor East, Australia) and 0.1% Tween-20 in Tris-buffered saline for 1 h and then incubated with primary antibodies (dilution 1: 1000–5000) overnight at 4 °C. The primary antibodies recognized the following proteins: fatty acid synthase (FAS, Santa Cruz, CA, USA), stearoyl-CoA desaturase-1 (SCD-1, Santa Cruz, CA, USA), sterol regulatory element-binding protein 1 (SREBP-1, Santa Cruz, CA, USA), peroxisome proliferator-activated receptor α (PPARα, Santa Cruz, CA, USA), AMP-activated protein kinase (AMPK, Cell Signaling, Danvers, MA, USA), phospho-AMPK (Cell Signaling), acetyl-CoA carboxylase (ACC, Cell Signaling), phospho-ACC (Cell Signaling), nuclear factor erythroid-2-related factor 2 (Nrf2, Santa Cruz, CA, USA), heme oxygenase 1 (HO-1, Santa Cruz, CA, USA), and β-actin (Santa Cruz, CA, USA). The membrane was incubated at room temperature for 1 h with peroxidase-conjugated secondary antibody (dilution 1:5000, Vector Laboratories, Burlingame, CA, USA), and proteins were detected using the Westar ETA C 2.0 substrate (Cyanagen, Bologna, Italy). 

### 2.5. Histological Analysis

Liver tissues were fixed with paraformaldehyde, washed, dehydrated, and embedded in paraffin. Then, paraffin blocks were prepared and sectioned using a microtome to prepare tissue slices. The serial paraffin sections (7 µm) were added to the xylene, washed through a hydration process, and then underwent hematoxylin and eosin staining. Stained tissue was dehydrated and enclosed with Canada balsam. For immunohistochemistry (IHC), staining for TNFα, paraffin sections were placed in xylene, underwent a hydration process and were washed with distilled water. Then, the tissue was reacted with 0.01 M sodium citrate buffer (pH 6.0; Sigma, St. Louis, MO, USA) and was then reacted with 0.3% H_2_O_2_ (Sigma) at room temperature and washed with distilled water. After serial washing with 0.1% PBS-T (1 × PBS/0.1% Tween 20 (Amresco, Solon, OH, USA)) and one wash with PBS, 1.5% normal goat serum was treated to the block at room temperature, which was then incubated overnight at 4 °C with TNF-α antibody (Life technologies, Carlsbad, CA, USA) diluted at 1:500. After the reaction was finished, tissues were washed with 0.1% PBS-T and once with PBS, and were left to react at room temperature with a horseradish-peroxidase-conjugated secondary antibody (biotinylated anti-Rabbit IgG) of Vectastain ABC kit (Vector Laboratories, Burlingame, CA, USA). The tissues were then washed with PBS-T and once with PBS, reacted at room temperature using Avidin–Biotin Complex (ABC) reagent, washed again with 0.1% PBS-T and PBS, and stained with the DAB peroxidase substrate kit (Vector Laboratories). When the color development was finished, it was washed with 1 × PBS, washed with distilled water, nuclear stained with Hematoxylin, washed with water again, and dehydrated. After that, it was enclosed with Canada balsam. The histological changes were observed under a microscope (DM500; Leica, Wetzlar, Germany).

### 2.6. RNA Extraction, Library, and Sequencing

Total RNAs were extracted from the liver tissues of the HF and SQE groups (*n* = 3) using QIAzol lysis reagent (Qiagen, Hilden, Germany), which were subsequently column-purified with a RNeasy mini kit (Qiagen). The RNA concentration and integrity of each sample were measured using an Agilent 2100 Bioanalyzer (Agilent, Santa Clara, CA, USA). A library was independently prepared with 1 µg of total RNA for each sample by the Illumina TruSeq Stranded mRNA Sample Prep Kit (Illumina Inc., San Diego, CA, USA). The libraries were quantified using KAPA Library Quantification kits for Illumina Sequencing platforms according to the qPCR Quantification Protocol Guide (Kapa Biosystems, Wilmington, MA, USA) and qualified using the TapeStation D1000 ScreenTape (Agilent Technologies). Indexed libraries were then submitted to Illumina NovaSeq (Illumina, Inc., San Diego, CA, USA) and paired-end (2 × 100 bp) sequencing was performed by Macrogen Inc. (Seoul, Korea).

### 2.7. mRNA-Seq Data and Gene Expression Level

The raw reads from the sequencer were preprocessed to remove low-quality and adapter sequences before analysis, and the processed reads were aligned to *Rattus norvegicus* (*rn6*) using HISAT v2.1.0 [26]. Transcript assembly and abundance estimation was performed using StringTie [27,28]. After alignment, StringTie v1.3.4d was used to assemble aligned reads into transcripts and estimate their abundance. The relative abundance of genes was measured in “Read Count” using StringTie. The statistical analysis to determine differentially expressed genes was performed using the estimates of abundance for each gene in the samples. Genes with Read Count values more than zero in the samples were excluded. To facilitate a log2 transformation, 1 was added to each Read Count value of filtered genes. Filtered data were log2-transformed and subjected to TMM normalization. Statistical significance of the differential expression data was determined using the “exactTest” function in edgeR and fold change, in which the null hypothesis was that no difference existed among groups. The false discovery rate (FDR) was controlled by adjusting the *p*-value using the Benjamini–Hochberg algorithm. For the DEG set, hierarchical clustering analysis was performed using complete linkage and Euclidean distance as a measure of similarity. Gene enrichment, functional annotation analysis, and pathway analysis for the significant gene list were performed based on gProfiler (https://biit.cs.ut.ee/gprofiler/orth), and Kyoto Encyclopedia of Genes and Genomes (KEGG) pathway (http://www.kegg.jp/kegg/). Gene set enrichment analysis (GSEA) was performed using default parameters in Molecular Signatures Data base version 4.1 (MsigDB 7.0).

### 2.8. Statistical Analysis

All statistical analyses were performed using SPSS v21 software (SPSS Inc.; Chicago, IL, USA). All data from experiments were expressed as mean ± SD and analyzed by one-way analysis of variance (ANOVA) with Tukey’s multiple comparisons on raw data reads. We considered a *p*-value < 0.05 to be statistically significant.

## 3. Results

### 3.1. Effect of SQE on Changes in Body and Organ Weight

We measured the changes in body weight among the experimental groups during the 16-week experimental period (Table 1). Initial weights were not significantly different among the experimental groups. The total weight gains in the high-carbohydrate-diet-fed rats (HC group) and high-fructose-diet-fed rats (HF group) were not significantly different. However, the high-fructose-diet-fed rats supplemented with SQE (SQE group) showed a reduction in body weight gain (329.00 ± 27.17 g) compared with the HF group (380.25 ± 28.17 g) fed the high-fructose diet only (*p* < 0.05). The total food intake in the HF and SQE groups did not differ significantly, but the intake of drinking water showed a significant difference (Table 1). The food efficiency ratio decreased significantly in the SQE group compared with the HC and HF groups. The weights of the liver, spleen, and epididymal fat mass in the SQE group were significantly lower than the HF group. These results suggest that SQE supplementation reduced the increase in body weight and tissue weights of the liver and spleen in high-fructose-diet-fed rats.

### 3.2. Effect of SQE on Serum Lipid Profiles

**Figure 1:**
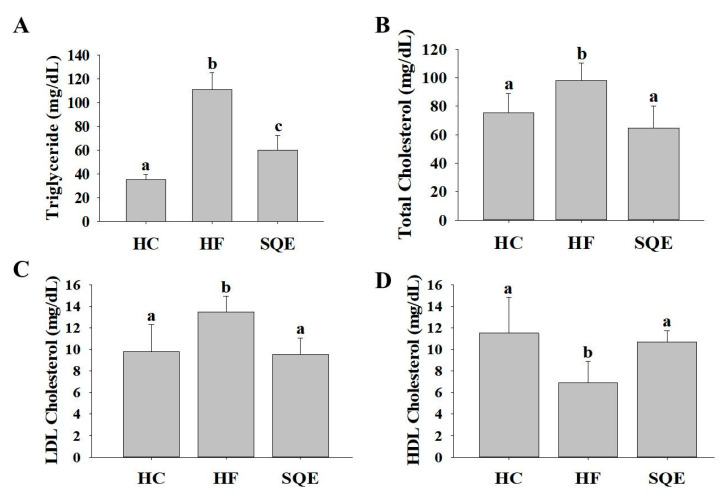
Effect of SQE supplementation on serum lipid levels in HF-diet-fed rats: (**A**) Serum triglyceride levels; (**B**) total cholesterol levels; (**C**) LDL-cholesterol levels; (**D**) HDL-cholesterol levels. Data are given as mean ± SD (*n* = 7). Different letters in each assay indicate significant differences among the HC, HF, and SQE groups by Tukey’s multiple range test (*p* < 0.05). HC, 46% carbohydrate diet; HF, 60% fructose diet; SQE, 60% fructose diet with SQE (500 mg/kg of BW) in drinking water. BW, body weight; HC, high-carbohydrate-diet-fed rats; HF, high-fructose-diet-fed rats; SQE: the high-fructose-diet-fed rats supplemented with *S. quelpaertensis* leaf extract; LDL-C, low-density lipoprotein cholesterol; HDL-C, high-density lipoprotein cholesterol, BW, body weight.

To evaluate the effects of SQE supplementation on lipid metabolic dysfunction in high-fructose-diet-fed rats, we investigated the serum lipid profiles in each experimental group after 16 weeks (Figure 1). The levels of triglycerides (TG), total cholesterol (TC), and low-density lipoprotein cholesterol (LDL-C) in the HF group were significantly higher than in the HC group, while the level of high-density lipoprotein cholesterol (HDL-C) was significantly lower compared with the HC group. These results indicate that the high-fructose diet induced dyslipidemia. Compared with the HF group, the SQE group exhibited significantly lower levels of TG, TC, and LDL-C, while the HDL-C level was significantly higher. These findings indicate that SQE supplementation improved the serum lipid profiles in the high-fructose-diet-fed rats.

### 3.3. Effects of SQE on Glucose Homeostasis

**Figure 2:**
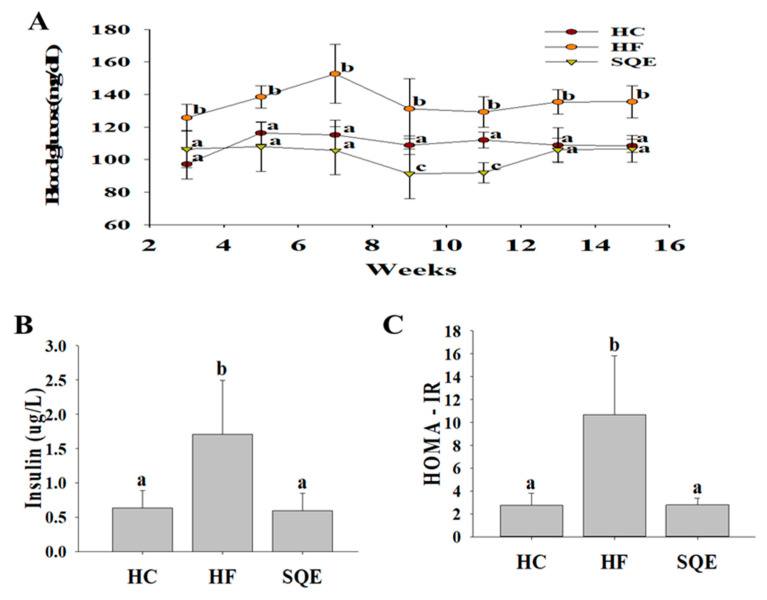
Effect of SQE supplementation on glucose homeostasis in HF-diet-fed rats: (**A**) Fasting blood glucose profile over 16 weeks; (**B**) serum insulin levels after 16 weeks; (**C**) HOMA-IR index after 16 weeks. Data are given as mean ± SD (*n* = 7). Different letters in each assay indicate significant differences among the HC, HF, and SQE groups by Tukey’s multiple range test (*p* < 0.05). HC, 46% carbohydrate diet; HF, 60% fructose diet; SQE, 60% fructose diet with SQE (500 mg/kg of BW) in drinking water. BW, body weight; HC, high-carbohydrate-diet-fed rats; HF, high-fructose-diet-fed rats; SQE, the high-fructose-diet-fed rats supplemented with *S. quelpaertensis* leaf extract; HOMA-IR, homeostasis model assessment of insulin resistance.

**Figure 3:**
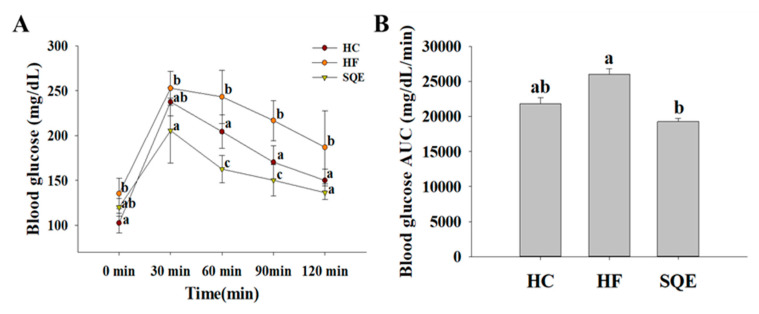
Effect of SQE supplementation on glucose tolerance by OGTT in HF-diet-fed rats: (**A**) Oral glucose tolerance test (OGTT); (**B**) the area under the curve (AUC). Data are given as mean ± SD (*n* = 7). Different letters in each assay indicate significant differences among the HC, HF, and SQE groups by Tukey’s multiple range test (*p* < 0.05). HC, 46% carbohydrate diet; HF, 60% fructose diet; SQE, 60% fructose diet with SQE (500 mg/kg of BW) in drinking water. BW, body weight; HC, high-carbohydrate-diet-fed rats; HF, high-fructose-diet-fed rats; SQE, the high-fructose-diet-fed rats supplemented with *S. quelpaertensis* leaf extract.

To assess the effects of SQE supplementation on glucose homeostasis in high-fructose-diet-fed rats, we measured their fasting blood glucose profiles over 16 weeks. As shown in Figure 2A, the fasting blood glucose levels in the HF group compared with the HC group were increased from 3 weeks after the initiation of the high-fructose diet and remained at a high level until 16 weeks. SQE supplementation significantly reduced the fasting blood glucose levels in high-fructose-diet-fed rats (Figure 2A). After 16 weeks, serum insulin levels (Figure 2B) and the homeostasis model assessment of insulin resistance (HOMA-IR) index (Figure 2C) in the SQE group were significantly lower compared with the HF group. These results indicate that SQE supplementation improved the insulin resistance induced by the high-fructose diet. We also performed an oral glucose tolerance test (OGTT) on individuals among each experimental group. As shown in Figure 3, the HF group induced more severe glucose tolerance than the HC group. However, SQE supplementation significantly ameliorated the glucose tolerance induced by the high-fructose diet.

### 3.4. Effects of SQE on Liver Function

**Figure 4:**
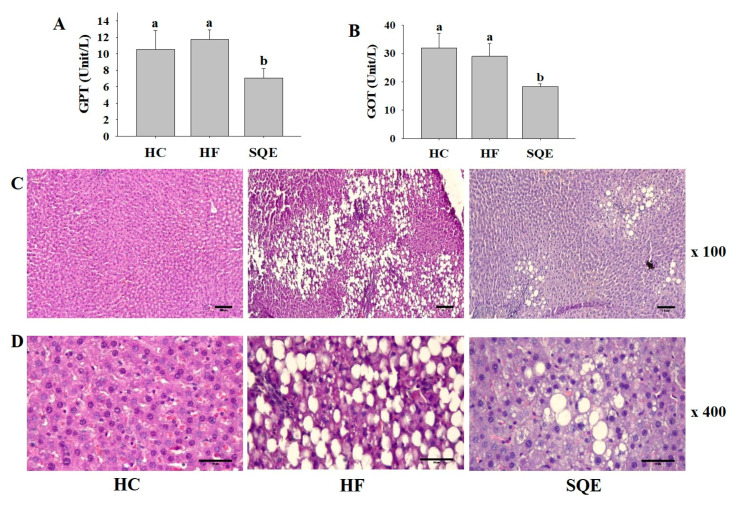
Effect of SQE supplementation on liver function in HF-diet-fed rats: (**A**) Glutamic pyruvic transaminase (GPT) and (**B**) glutamic oxaloacetic transaminase (GOT) levels. Data are given as mean ± SD (*n* = 7). Different letters in each assay indicate significant differences among the HC, HF, and SQE groups by Tukey’s multiple range test (*p* < 0.05). (**C**,**D**) Hematoxylin and eosin staining on paraffin sections at ×100 (**C**) and ×400 (**D**) magnification with scale bars of 200 μm and 50 μm. (HC, 46% carbohydrate diet; HF, 60% fructose diet; SQE, 60% fructose diet with SQE (500 mg/kg of BW) in drinking water. BW, body weight; HC, high-carbohydrate-diet-fed rats; HF, high-fructose-diet-fed rats; SQE, the high-fructose-diet-fed rats supplemented with *S. quelpaertensis* leaf extract.

**Figure 5:**
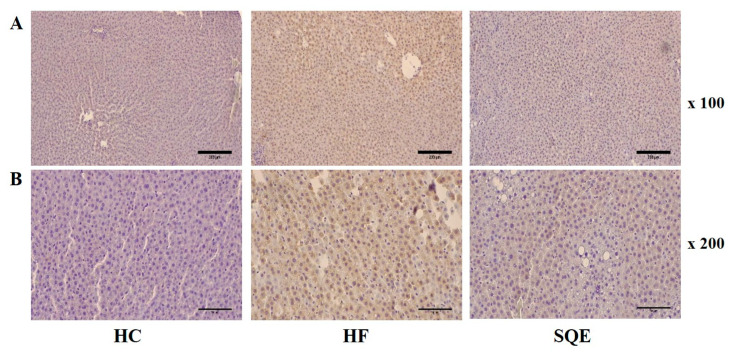
Effect of SQE supplementation on TNFα expression in liver tissues of HF-diet-fed rats. IHC staining against TNFα in liver paraffin sections at ×100 (**A**) and ×200 (**B**) magnification, with scale bars of 200 μm and 100 μm. HC, 46% carbohydrate diet; HF, 60% fructose diet; SQE, 60% fructose diet with SQE (500 mg/kg of BW) in drinking water. BW, body weight; HC, high-carbohydrate-diet-fed rats; HF, high-fructose-diet-fed rats; SQE, the high-fructose-diet-fed rats supplemented with *S. quelpaertensis* leaf extract.

SQE supplementation significantly lowered the increase in serum glutamic oxaloacetic transaminase (GOT) and glutamic pyruvic transaminase (GPT) levels induced by the high-fructose diet (Figure 4A,B), indicating its protective effect on liver function. We performed a histological analysis of live tissues for each group. Hematoxylin and eosin staining revealed that the liver tissues of the HF group progressed to NAFLD by accumulating larger lipid droplets than the HC group. However, the liver tissues of the SQE group had a reduced size and number of lipid droplets (Figure 4C,D), which is consistent with the observation of the liver weight indices shown in Table 1. We then assessed the effect of SQE supplementation on high-fructose-diet-induced metabolic inflammation by immunohistochemistry (IHC) staining against tumor necrosis factor α (TNFα), a proinflammatory mediator in liver tissues. IHC staining clearly showed that SQE supplementation reduced the expression of TNFα. These results indicate that SQE supplementation can ameliorate high-fructose-diet-induced metabolic inflammation (Figure 5A,B).

### 3.5. Effects of SQE on Protein Expression in Liver Tissue

**Figure 6:**
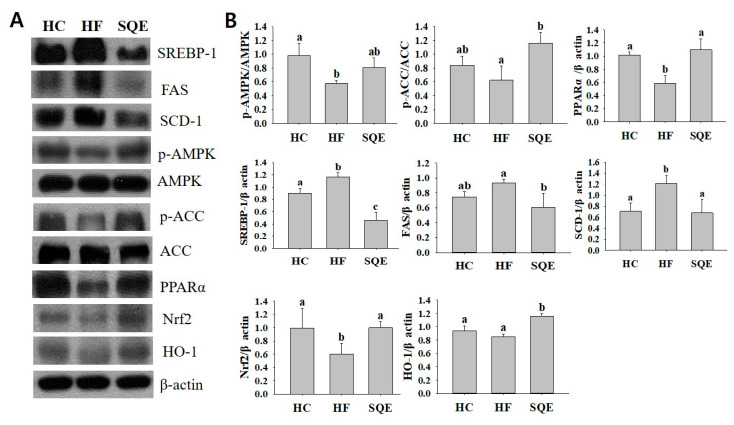
Effect of SQE on protein expressions in liver tissues of HF-diet-fed rats: (**A**) Protein levels were determined by Western blotting, and the data shown are representative of three experiments; (**B**) relative band intensity was determined by densitometry and normalized with β-actin. Results are expressed as the mean ± SD (*n* = 4). Different letters in each assay indicate significant differences among the HC, HF, and SQE groups by Tukey’s multiple range test (*p* < 0.05). HC, 46% carbohydrate diet; HF, 60% fructose diet; SQE, 60% fructose diet with SQE (500 mg/kg of BW) in drinking water. BW, body weight; HC, high-carbohydrate-diet-fed rats; HF, high-fructose-diet-fed rats; SQE, the high-fructose-diet-fed rats supplemented with *S. quelpaertensis* leaf extract.

To explore the underlying molecular mechanism of the beneficial effects of SQE in liver tissue, we performed Western blot analysis to investigate the effects of SQE on the expressions of proteins involved in lipid metabolism and antioxidant response. The expression of sterol regulatory element-binding protein 1 (SREBP-1), fatty acid synthase (FAS), and stearoyl-CoA desaturase-1 (SCD-1), which promote DNL in liver tissues, were significantly higher in the HF group compared with the HC group, indicating that the high-fructose diet increased the DNL in liver tissue (Figure 6). However, SQE supplementation lowered the expression of SREBP-1, FAS, and SCD-1 in liver tissue. SQE supplementation increased the phosphorylation of AMP-activated protein kinase (AMPK) and acetyl-CoA carboxylase (ACC), which are involved in the oxidation of fatty acids, while it upregulated the expression of antioxidant proteins, such as peroxisome proliferator-activated receptor α (PPARα), nuclear factor erythroid-2-related factor 2 (Nrf2), and heme oxygenase 1 (HO-1), which play key roles in responding to oxidative stress (Figure 6A,B). These results indicate that SQE supplementation ameliorated the hepatic oxidative stress induced by the high-fructose diet.

### 3.6. Effect of SQE on Liver Transcriptome Profiles

To explore how the SQE supplementation affects whole gene expressions in high-fructose-diet-fed rats, we further performed RNA-Seq to analyze the liver transcriptome profiles of the HF and SQE groups (*n* = 3). As shown in Table 2, the guanine–cytosine contents averaged between 49.00% and 49.50% in the HF and SQE groups, while Q30 content was at least 95.58%, indicating good sequencing quality. We obtained approximately 63,132,988–87,240,986 high-quality clean reads from the rats of the HF and SQE groups. Among them, more than 95.58% of the clean reads in each sample were uniquely mapped to *Rattus norvegicus*, indicating that the quality of all the libraries was suitable for subsequent analysis. 

**Figure 7:**
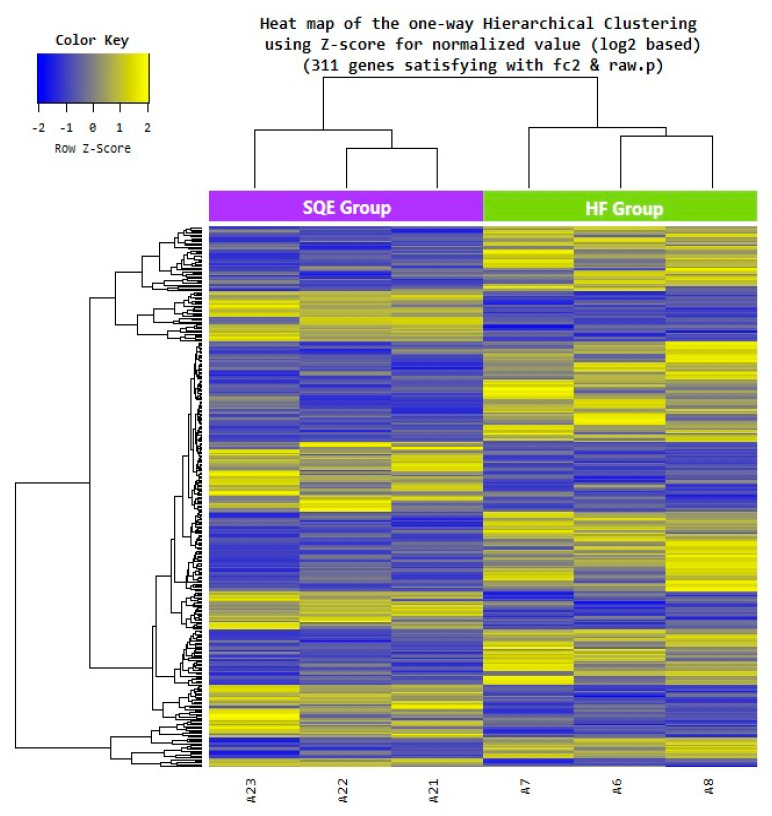
Heat map of the one-way hierarchical clustering using the Z-score for the normalized value from 311 differentially expressed genes (DEGs) between the SQE group and HF group. HF, high-fructose-diet-fed rats; SQE, the high-fructose-diet-fed rats supplemented with *S. quelpaertensis* leaf extract.

**Figure 8:**
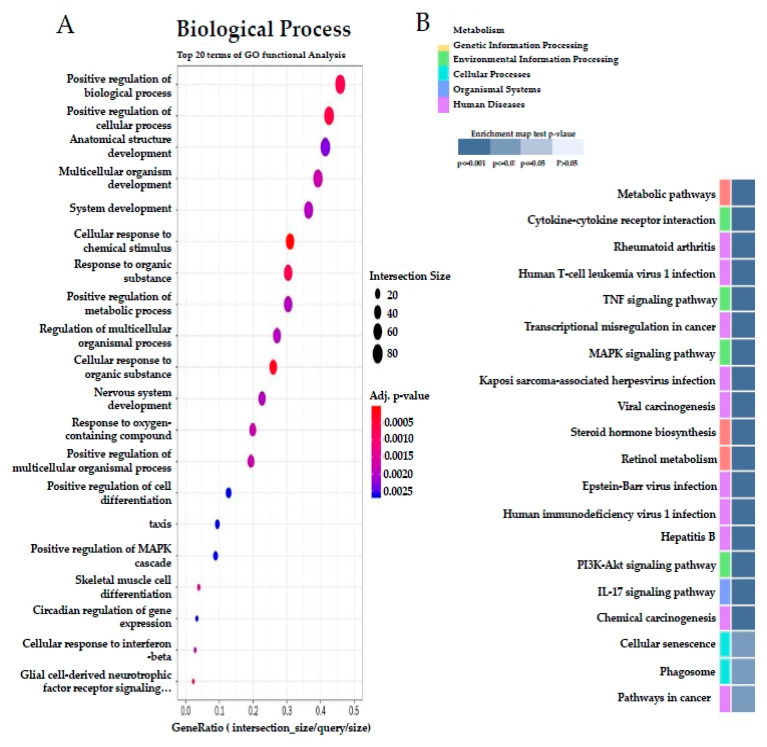
Effects of SQE on liver transcriptome profiles in HF-diet-fed rats. (**A**) Gene ontology (GO) analysis: Top twenty terms of GO function analysis in “biological process” are shown. (**B**) Kyoto Encylopedia of Genes and Genomes (KEGG) pathway analysis: Top twenty terms of KEGG pathway are shown. *p* value shown in modified Fisher‘s exact test.

**Figure 9:**
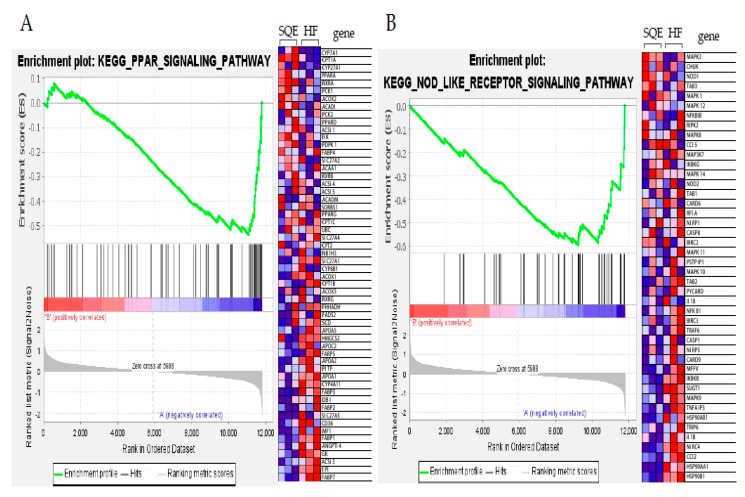
Gene set enrichment analysis (GSEA) plots depict the differentially expressed genes (DEGs) identified between the HF and SQE groups. (**A**) PPAR signaling pathway categories, (**B**) NOD-like receptor signaling pathway. HF, 60% fructose diet; SQE, 60% fructose diet with SQE (500 mg/kg of BW) in drinking water. HF, high-fructose-diet-fed rats; SQE, the high-fructose-diet-fed rats supplemented with *S. quelpaertensis* leaf extract.

To identify the differentially expressed genes (DEGs) between the HF and SQE groups, gene expression data from each group were compared. Among the 15,382 detected genes, we extracted 311 DEGs comprising 126 upregulated and 185 downregulated genes, in SQE vs. HF groups (fold change ≥2, *p* < 0.05, Table S2). Hierarchical clustering analysis of these DEGs showed similar gene expression patterns among individuals within the HF or SQE groups rather than between groups (Figure 7). To understand the signatures of these DEGs under SQE supplementation, Gene Ontology (GO) and Kyoto Encyclopedia of Genes and Genomes (KEGG) pathway analyses were performed. According to the GO analysis, the four most enriched GO terms (biological processes) by SQE supplementation were cellular response to chemical stimulus, cellular response to organic substance, positive regulation of cellular process, and positive regulation of biological process (Figure 8A). KEGG pathway analysis revealed that SQE supplementation significantly enriched the pathways involved in metabolism, environmental information processing, cellular processes, organismal systems, and human diseases (Figure 8B). In addition, we performed gene set enrichment analysis (GSEA) using DEGs identified between the HF and SQE groups. GSEA analysis showed that SQE regulated the genes in the PPAR signaling pathway and the nucleotide-binding oligomerization domain (NOD)-like receptor signaling pathway (Figure 9). Consistent with the changes in the protein expressions between the HF and SQE groups, the KEGG pathway analysis demonstrated that SQE supplementation modulated the genes related to carbohydrate and lipid metabolism and environmental information processing such as the MAPK signaling pathway, the TNF signaling pathway, the PI3K-Akt signaling pathway, the AMPK signaling pathway, and the interleukin-17 signaling pathway. These results indicate that SQE supplementation can ameliorate insulin resistance, dyslipidemia, and hepatic steatosis of rats by regulating the genes relevant to metabolic dysfunction in high-fructose-diet-fed rats.

## 4. Discussion

In this study, we investigated the effects of SQE on insulin resistance, hyperlipidemia, and NAFLD in high-fructose-diet-fed rats. The average dietary intake of SQE is still not known. However, several animal studies have reported the beneficial effects of oral supplementation of SQE (100–300 mg/day/kg of body weight) [19,20,29,30]. Based on this information, we implemented a supplementation dose of SQE of 500 mg/day/per kg of body weight via drinking water. This dose was believed to be in an effective, nontoxic-side-effect-producing range. The chromatogram of SQE analyzed with HPLC-PDA indicates that SQE contains various phytochemicals including tricin and *p*-coumaric acid (Figure S1). Previously, these compounds were found to be beneficial for the improvement of hepatotoxicity and inflammation-associated colon carcinogenesis [30,31]. SQE also contains various flavonoids including tricin, *p*-coumaric acid, isoorientin, orientin, vitexin, and ferulic acid [32]. Thus, the beneficial effects of SQE in this animal model might be due to the synergistic actions exerted by these phytochemicals.

It has been reported that the chronic intake of a high-fructose diet might be a risk factor for the development of NAFLD, insulin resistance, and metabolic syndrome [33,34,35]. Here, we found that a 60% fructose intake for 16 weeks caused body weight gain and increases in fat and liver weights in rats. Further, high fructose consumption increased serum levels of TG, TC, and LDL-C and lowered the serum levels of HDL-C. These changes in serum parameters alter the energy metabolism in the liver and are risk factors for the development of diseases, such as NAFLD, insulin resistance, and metabolic syndrome [9]. In particular, the increased accumulation of free fatty acids, TG, and TC in mitochondria leads to liver damage and reactive oxygen species (ROS) formation mediated by tumor necrosis factor alpha (TNFα) and interleukin-6 (IL-6), which have been demonstrated to play an essential role in the pathopoiesis of NAFLD [36,37]. In this study, the SQE supplementation (500 mg/day/kg of BW) via drinking water reduced body weight gain and liver and spleen indices in the high-fructose-diet-fed rats. SQE supplementation reduced serum TC, TG, and LDL-C levels and increased HDL-C level in high-fructose-diet-fed rats. In addition to the improvements in serum lipid profiles, the histological examinations showed that SQE supplementation reduced the fat deposition of liver cells compared with the high-fructose-diet-fed rats without SQE treatment. 

SQE supplementation significantly reduced the expression of TNFα in liver tissue compared with high-fructose-diet-fed rats. Metabolic inflammation plays an essential role in the development of obesity and related metabolic diseases [38]. It is well-known that the overproduction of proinflammatory cytokines TNFα, IL-1β, IL-6, and MCP-1 might play a critical role in the development of insulin resistance and chronic inflammation in obese animals [39]. Excessive intake of fructose causes intestinal-barrier deterioration and endotoxaemia, which engages Toll-like receptor 4 to trigger TNF production by liver macrophages, thereby promoting DNL in both mouse and human hepatocytes [40]. It is interesting that *Sasa quelpaertensis* leaf extract regulates microbial dysbiosis by modulating the composition and diversity of the microbiota in dextran sulfate sodium-induced colitis mice [28]. Thus, it is suggested that SQE may have alleviated NAFLD in the high-fructose-diet-fed rats via its anti-inflammatory properties. 

Consistent with previous studies, the present study revealed that the high-fructose diet induced insulin resistance, as indicated by high levels of serum insulin and glucose, and high values of HOMA-IR. Furthermore, after glucose loading during OGTT, the glucose concentration was significantly higher in HF group rats. This result indicates that the ability of insulin to stimulate glucose disposal was dramatically impaired in rats of the HF group, which was related to insulin resistance after chronic fructose feeding. Insulin resistance is an important characteristic of NAFLD and type 2 diabetes. In contrast, SQE supplementation decreased serum insulin and glucose levels, and improved body insulin resistance in the rats of the HF group. This result suggests that SQE might play an important role in the prevention of NAFLD complicated with type 2 diabetes and metabolic syndrome.

AMPK plays an important role in maintaining energy homeostasis including hepatic lipid metabolism. It serves as a metabolic master switch in response to alterations in cellular energy charge [41]. SQE supplementation not only increased the phosphorylation of AMPK and ACC, but also increased the expression of PPARα. This suggests that SQE supplementation modulated lipid synthesis, lipolysis, and fatty acid oxidation through AMPK-driven pathways such as inactivation of ACC and upregulation of PPARα [42]. SQE supplementation downregulated SREBP-1 and its target genes FAS and SCD-1. The precursor to SREBP-1, which is synthesized in the endoplasmic reticulum membrane, undergoes proteolytic processing to release the transcriptionally active N-terminal domain, which is subsequently translocated into the nucleus and promotes its target genes [43]. We found that SQE supplementation decreased the expression of FAS, ACC, and SCD-1 through the AMPK and PPARα-mediated SREBP-1 pathway in liver tissue. Furthermore, SQE supplementation increased the expression of Nrf2 and its target HO-1 in liver tissue. Nrf2 is a transcription factor that binds antioxidant response elements (AREs) in the regulatory regions of target genes, and it acts as a main player in the inducible expression of cellular defense enzymes. This suggests that SQE supplementation could have a significant protective effect against high-fructose-diet-induced oxidative stress, which is associated with the pathogenesis of liver injury and fatty liver [34,43]. 

The transcriptome analysis indicated that SQE supplementation mainly enriched the genes that participate in metabolic pathways including lipid metabolism, retinol metabolism, and metabolism of xenobiotics by cytochrome P450. Western blot analysis on the lipid metabolism-related proteins demonstrated that SQE supplementation increased fatty acid oxidation but decreased de novo lipogenesis in the liver tissue of high-fructose-diet-fed rats. In addition, KEGG pathway analysis showed that SQE supplementation affected the MAPK signaling pathway, TNF signaling pathway, PI3K-Akt signaling pathway, and IL-17 signaling pathway, among others. GSEA analysis revealed that SQE supplementation significantly altered the genes involved in the PPAR signaling pathway such as *CYP7A1*, *CTP1A*, *CYP27A1*, *PPARA*, *FADS1*, *SCD*, *RXRA*, and *PCK1*; NOD-like receptor signaling pathway, such as *IL8*, *MAPK3*, *NOD1*, *NFKBIB*, *IL1B*, and *PIPK2*. Since these pathways are relevant to metabolic dysfunctions, such as the insulin resistance and metabolic inflammation induced in the high-fructose-diet-fed rats in this study, we hypothesize that SQE supplementation could alleviate hepatic inflammation and insulin resistance in high-fructose-diet-fed rats. 

In conclusion, we have demonstrated, for the first time, that SQE ameliorates insulin resistance, dyslipidemia, and NAFLD in high-fructose-fed rats. These effects are mostly exerted by downregulating lipogenesis and upregulating fatty acid oxidation, as well as by preventing inflammatory response and oxidative stress. The transcriptome analysis confirmed that SQE enriched the genes that mediate the regulation of the lipid metabolism pathway, insulin signaling pathway, and MAPK signaling pathway. Thus, *Sasa quelpaertensis* leaf extract could have potential as a functional food ingredient to ameliorate lifestyle-related metabolic disorders.

## Figures and Tables

**Table 1:** Effect of *Sasa quelpaertensis* leaf extract (SQE) supplementation on body weight, food intake, water intake, food efficiency ratio, and organ indices in high-fructose-diet-fed (HF group) rats.

Groups	HC	HF	SQE
Initial body weight (g)	322.50 ± 17.77	322.87 ± 22.56	309.79 ± 12.87
Final body weight (g)	699.63 ± 70.02 ^a^	703.13 ± 42.48 ^a^	638.75 ± 35.75 ^b^
Weight gain (g)	377.12 ± 57.08 ^a^	380.25 ± 28.17 ^a^	329.00 ± 27.17 ^b^
Total food intake for 16 weeks (kcal/g)	11,600.82 ± 79.22 ^a^	16,520.70 ± 148.46 ^b^	16,030.99 ± 113.54 ^b^
Total water intake for 16 weeks (mL)	5197.25 ± 27.30 ^a^	6597.06 ± 37.49 ^b^	7476.96 ± 95.24 ^c^
Food efficiency ratio (%)	9.31 ± 1.29 ^a^	8.43 ± 0.49 ^a^	7.28 ± 0.57 ^b^
Liver weight (g)	14.93 ± 2.63 ^a^	20.15 ± 4.97 ^b^	15.11 ± 1.78 ^a^
Spleen weight (g)	1.01 ± 0.19 ^a^	1.00 ± 0.14 ^a^	0.78 ± 0.09 ^b^
Kidney weight (g)	1.69 ± 0.17 ^a^	2.08 ± 0.20 ^b^	1.87 ± 0.20 ^ab^
Testis weight (g)	1.70 ± 0.11	1.82 ± 0.11	1.61 ± 0.38
Epididymal fat (g)	0.43 ± 0.11 ^a^	0.57 ± 0.11 ^b^	0.42 ± 0.06 ^a^

Data are given as mean ± SD (*n* = 7). Different letters in each column indicate significant difference from each other among the HC, HF, and SQE groups by Tukey’s multiple range test (*p* < 0.05). HC, 46% carbohydrate diet; HF, 60% fructose diet; SQE, 60% fructose diet with SQE (500 mg/kg of BW) in drinking water; BW, body weight; HC, high-carbohydrate-diet-fed rats; HF, high-fructose-diet-fed rats; SQE, the high-fructose-diet-fed rats supplemented with *S. quelpaertensis* leaf extract.

**Table 2:** Summary of sequencing read alignment to the reference genome.

Sample	Total Reads	GC Contents (%)	Q30 (%)	Processed Reads	Mapped Reads	Unmapped Reads
HF-1	63,992,692	49.00	95.58	63,992,692	62,592,420 (97.81%)	1,400,273 (2.19%)
HF-2	63,132,988	49.18	95.80	63,132,988	61,846,383 (97.96%)	1,286,605 (2.04%)
HF-3	75,518,180	49.50	95.59	75,518,180	73,953,123 (97.93%)	1,565,057 (2.07%)
SQE-1	83,369,682	49.13	95.71	83,396,682	81,437,777 (97.65%)	1,958,905 (2.35%)
SQE-2	64,484,030	49.11	95.83	64,484,030	63,011,809 (97.72%)	1,472,221 (2.28%)
SQE-3	87,240,986	49.30	95.74	87,240,986	85,412,156 (97.90%)	1,828,830 (2.1%)

## Supplementary Materials

The following are available online at https://www.mdpi.com/2072-6643/12/12/3762/s1: Figure S1. HPLC-PDA chromatogram of SQE, Table S1. Composition of experimental diets (g/kg), Table S2. List of differentially expressed genes (DEGs) in SQE group vs. high fructose (HF) diet group.
