# Supplementary material for: Sasa quelpaertensis Leaf Extract Ameliorates Dyslipidemia, Insulin Resistance, and Hepatic Lipid Accumulation in High-Fructose-Diet-Fed Rats
Source: Nutrients. 2020 Dec 7;12(12):3762. doi: 10.3390/nu12123762 (PMC7762336; doi:10.3390/nu12123762)
Supplement: Supplementary file 1 [file nutrients-12-03762-s001.pdf]

Supplementary Materials

Table S1. Composition of experimental diets (g/kg)

Ingredients	HC	HF
Casein	200 g (714 kcal)	207 g (828 kcal)
DL-methionine	3 g (12 kcal)	3 g (12 kcal)
Corn starch	397.49 g (1431 kcal)	0
Fructose	0	600 g (2400 kcal)
Lard	0	50 g (450 kcal)
Maltodextrin	62.514 g (250 kcal)	0
Sucrose	0	0
Soybean oil	70 g (630 kcal)	0
Cellulose	219.49 g (0 kcal)	79.81 g (0 kcal)
AIN 93G Mineral Mix	35 g (0 kcal)	0
AIN 93G Vitamin Mix	10 g (40 kcal)	0
Choline bitartrate	2.5 g (0 kcal)	0
TBHQ	0.01 g (0 kcal)	0
Rogers-Harper Mineral Mix	0	50 g (0 kcal)
Zinc carbonate	0	0.04 g (0 kcal)
Vitamin Mix 40060	0	10 g (40 kcal)
Total	1000 g (3077 kcal)	1000 g (3730 kcal)

HC (high carbohydrate), 46% carbohydrate diet modified based on AIN-93G (Harlan Laboratories, Madison, WI, USA); HF (high fructose), 60% fructose diet based on regular diet (Envigo, Madison, WI, USA).

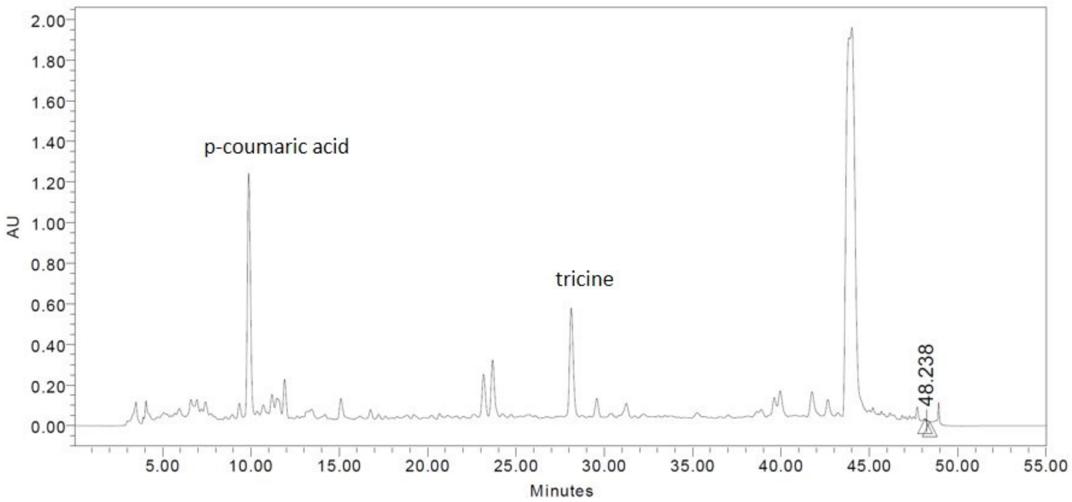

Figure S1. HPLC-PDA chromatogram of SQE. Retention time and indicator compounds are shown. SQE was analyzed using XBridge BEH C_{18} column (4.6 × 250 mm, 5 μm) in an analytical HPLC instrument (Water 2695 Alliance system) equipped with a photodiode array detector at 320 nm. Chromatographic separation was performed using a gradient elution at a flow rate of 0.8 mL/min with mobile phase for 0.5% acetic acid in distilled water (A)/0.5% acetic acid in acetonitrile (B) as follows: 0.0 min, 85.0% A and 15.0% B; 40.0 min, 57.5% A and 42.5% B; 40.1–45.0 min, 100% B; 45.0–55 min, 85.5% A. The separation was carried out at 40 °C, with a sample injection volume of 10 μL and a flow rate of 0.8 mL/min.

Table S2. List of differentially expressed genes in SQE group vs high fructose (HF) group

Gene_ID	Transcript_ID	Gene_Symbol	Description	gene_biotype	Protein_ID	Fold changes
24296	NM_012540, XM_006243150	Cyp1a1	cytochrome P450, family 1, subfamily a, polypeptide 1	protein_coding	NP_036672.2;XP_006243212.1	6.218034
24330	NM_012551	Egr1	early growth response 1	protein_coding	NP_036683.1	-2.675282
24516	NM_021835	Jun	Jun proto-oncogene, AP-1 transcription factor subunit	protein_coding	NP_068607.1	-2.514210
24517	NM_021836	Junb	JunB proto-oncogene, AP-1 transcription factor subunit	protein_coding	NP_068608.2	-2.456164
24522	NM_052798, XM_006246227, XM_008767660, XM_008767661	Zfp354a	zinc finger protein 354A	protein_coding	NP_434685.1;XP_006246289.1;XP_008765882.1;XP_008765883.1	10.417768
24596	NM_012610, XM_017597000, XM_017597001	Ngfr	nerve growth factor receptor	protein_coding	NP_036742.2;XP_017452489.1;XP_017452490.1	-2.602373
24626	NM_017031	Pde4b	phosphodiesterase 4B	protein_coding	NP_058727.2	-2.428865
24684	NM_001034111, NM_012630, XM_017590635	Prlr	prolactin receptor	protein_coding	NP_001029283.1;NP_036762.1;XP_017446124.1	-2.569681
24716	NM_001110099, NM_012643, XM_017592470, XM_017592471	Ret	ret proto-oncogene	protein_coding	NP_001103569.1;NP_036775.2;XP_017447959.1;XP_017447960.1	-2.879924
24723	NR_046239	Rn45s	45S pre-ribosomal RNA	rRNA	.	-22.410016
24770	NM_031530	Ccl2	C-C motif chemokine ligand 2	protein_coding	NP_113718.1	-2.721859

25083	NM_012739	Adra2a	adrenoceptor alpha 2A	protein_coding	NP_036871.3	3.113242
25148	NM_017086, XM_006252240	Egr3	early growth response 3	protein_coding	NP_058782.1;XP_006252302.1	-2.291442
25415	NM_012932, XM_006251084	Crmp1	collapsin response mediator protein 1	protein_coding	NP_037064.1;XP_006251146.1	2.620569
25426	NM_012940, XM_017594045, XM_017594046	Cyp1b1	cytochrome P450, family 1, subfamily b, polypeptide 1	protein_coding	NP_037072.1;XP_017449534.1;XP_017449535.1	-2.383372
25428	NM_012942	Cyp7a1	cytochrome P450, family 7, subfamily a, polypeptide 1	protein_coding	NP_037074.1	3.657290
25505	NM_001142367, NM_001285415, NM_012997	P2rx1	purinergic receptor P2X 1	protein_coding	NP_001135839.1;NP_001272344.1;NP_037129.1	-2.542189
29194	NM_017127, XM_006230707, XM_006230708, XM_008760090, XM_017588852, XM_017588853	Chka	choline kinase alpha	protein_coding	NP_058823.1;XP_006230769.1;XP_006230770.1;XP_008758312.1;XP_017444341.1;XP_017444342.1	-2.227074
29316	NM_017161	Adora2b	adenosine A2B receptor	protein_coding	NP_058857.1	-3.923243
29441	NM_031576	Por	cytochrome p450 oxidoreductase	protein_coding	NP_113764.1	2.182538
29455	NM_019216	Gdf15	growth differentiation factor 15	protein_coding	NP_062089.1	-2.252260
29480	NM_017214	Rgs4	regulator of G-protein signaling 4	protein_coding	NP_058910.1	-2.058442
29517	NM_001193568, NM_001193569, NM_019232, XM_006227723	Sgk1	serum/glucocorticoid regulated kinase 1	protein_coding	NP_001180497.1;NP_001180498.1;NP_062105.2;XP_006227785.1	-2.095898
29619	NM_017259	Btg2	BTG family, member 2	protein_coding	NP_058955.1	-2.644351

29657	NM_024362, XM_006230029, XM_006230030, XM_017589065, XM_017589066, XM_017589067, XM_017589068, XM_017589069, XM_017589070, XM_017589071	Arntl	aryl hydrocarbon receptor nuclear translocator-like	protein_coding	NP_077338.2;XP_006230091.1;XP_006230092.1;XP_017444554.1;XP_017444555.1;XP_017444556.1;XP_017444557.1;XP_017444558.1;XP_017444559.1;XP_017444560.1	-2.948863
29692	NM_031598, XM_006239146	Pla2g2a	phospholipase A2 group IIA	protein_coding	NP_113786.3;XP_006239208.1	-2.299710
29710	NM_019266	Scn8a	sodium voltage-gated channel alpha subunit 8	protein_coding	NP_062139.2	2.363178
54300	NM_019345, XM_008772323, XM_008772324, XM_008772325, XM_008772326, XM_008772327, XM_008772328	Slc12a3	solute carrier family 12 (sodium/chloride transporters), member 3	protein_coding	NP_062218.3;XP_008770545.1;XP_008770546.1;XP_008770547.1;XP_008770548.1;XP_008770549.1;XP_008770550.1	3.932854
56780	NM_001134901, NM_020072, XM_017595869, XM_017595870, XM_017595871	Acpp	acid phosphatase, prostate	protein_coding	NP_001128373.1;NP_064457.1;XP_017451358.1;XP_017451359.1;XP_017451360.1	-2.285176
57298	NM_020540	Gstm3	glutathione S-transferase mu 3	protein_coding	NP_065415.1	-2.026246
59109	NM_021589	Ntrk1	neurotrophic receptor tyrosine kinase 1	protein_coding	NP_067600.1	4.403724
60423	NM_031664	Slc28a2	solute carrier family 28 member 2	protein_coding	NP_113852.1	-2.178636

60577	NM_031672	Slc15a2	solute carrier family 15 member 2	protein_coding	NP_113860.2	5.721409
60629	NM_022206, XM_008771505	Unc5a	unc-5 netrin receptor A	protein_coding	NP_071542.1;XP_008769727.1	2.010986
65198	NM_031742, XM_006250491, XM_017598933	Kcnh1	potassium voltage-gated channel subfamily H member 1	protein_coding	NP_113930.1;XP_006250553.1;XP_017454422.1	2.201778
65984	NM_023104	Aacs	acetoacetyl-CoA synthetase	protein_coding	NP_075592.1	2.053666
79240	NM_024388, XM_006242356, XM_006242358, XM_017595130	Nr4a1	nuclear receptor subfamily 4, group A, member 1	protein_coding	NP_077364.2;XP_006242418.1;XP_006242420.1;XP_017450619.1	-2.208187
79251	NM_024399, XM_006246815, XM_006246817, XM_006246818, XM_017597543	Aspa	aspartoacylase	protein_coding	NP_077375.1;XP_006246877.1;XP_006246879.1;XP_006246880.1;XP_017453032.1	2.328696
80841	NM_030832	Fabp7	fatty acid binding protein 7	protein_coding	NP_110459.1	-2.311611
81503	NM_030845	Cxcl1	chemokine (C-X-C motif) ligand 1	protein_coding	NP_110472.1	-2.766409
81670	NM_031039	Gpt	glutamic--pyruvic transaminase	protein_coding	NP_112301.1	2.001633
83476	NM_031327	Cyr61	cysteine-rich, angiogenic inducer, 61	protein_coding	NP_112617.2	-3.491657
84395	NM_053380	Slc34a2	solute carrier family 34 member 2	protein_coding	NP_445832.1	-6.592417
84422	NM_053398	Gfra3	GDNF family receptor alpha 3	protein_coding	NP_445850.1	2.178610
113892	XM_006236798	Nat8f3	N-acetyltransferase 8 (GCN5-related) family	protein_coding	XP_006236860.1	3.968642

			member 3			
114090	NM_053633, XM_008772857, XM_017601530, XM_017601531	Egr2	early growth response 2	protein_coding	NP_446085.1;XP_008771079.2;XP_017457019.1;XP_017457020.1	-2.117107
114108	NM_053650, XM_006253119, XM_006253120	Pdlim3	PDZ and LIM domain 3	protein_coding	NP_446102.1;XP_006253181.1;XP_006253182.1	-2.977611
114122	NM_001170558, NM_001170559, NM_001170560, NM_053663	Vcan	versican	protein_coding	NP_001164029.1;NP_001164030.1;NP_001164031.1;NP_446115.1	-3.002808
114491	NM_053699	Cited4	Cbp/p300-interacting transactivator, with Glu/Asp-rich carboxy-terminal domain, 4	protein_coding	NP_446151.1	2.701477
114494	NM_053702, XM_006232266, XM_008760903	Ccna2	cyclin A2	protein_coding	NP_446154.3;XP_006232328.1;XP_008759125.1	-2.259163
114495	NM_053703	Map2k6	mitogen-activated protein kinase kinase 6	protein_coding	NP_446155.1	3.793457
115768	XM_008763803	Zfp37	zinc finger protein 37, transcript variant X1	protein_coding	XP_008762025.2	2.684012
115771	NM_053774, XM_006242861, XM_006242863, XM_008766138, XM_008766139	Usp2	ubiquitin specific peptidase 2	protein_coding	NP_446226.2;XP_006242923.1;XP_006242925.1;XP_008764360.1;XP_008764361.1	4.800462
116471	gene8188, id146797	Igkv28	.	.	.	-4.664054
116568	NM_053840	Ggt1	gamma-glutamyltransferase 1	protein_coding	NP_446292.2	2.334887
116597	NM_053848,	Opcml	opioid binding	protein_coding	NP_446300.1;XP_017450901.1;XP_01745	-2.512983

	XM_017595412, XM_017595413, XM_017595414, XM_017595415, XM_017595416, XM_017595417, XM_017595418		protein/cell adhesion molecule-like		0902.1;XP_017450903.1;XP_017450904.1;XP_017450905.1;XP_017450906.1;XP_017450907.1	
117033	NM_053963, XM_017595420	Mmp12	matrix metallopeptidase 12	protein_coding	NP_446415.2;XP_017450909.1	-2.394912
117271	NM_057130	Hrk	harakiri, BCL2 interacting protein	protein_coding	NP_476471.1	3.219076
117549	NM_057201	Gpr37	G protein-coupled receptor 37	protein_coding	NP_476549.1	-2.111426
117560	NM_057211	Klf9	Kruppel-like factor 9	protein_coding	NP_476559.1	2.205174
155192	NM_130414, XM_008764453	Abcg8	ATP binding cassette subfamily G member 8	protein_coding	NP_569098.2;XP_008762675.1	2.523936
170496	NM_130741	Lcn2	lipocalin 2	protein_coding	NP_570097.1	-2.822466
171352	NM_147206	Cyp3a9	cytochrome P450, family 3, subfamily a, polypeptide 9	protein_coding	NP_671739.2	2.471669
171393	NM_134376	Clstn3	calsyntenin 3	protein_coding	NP_599203.1	-2.920487
171493	NM_138504, XM_006255706, XM_006255707	Osgin1	oxidative stress induced growth inhibitor 1	protein_coding	NP_612513.1;XP_006255768.1;XP_006255769.1	-2.037585
192235	NM_001034028, NM_138867, XM_006242884, XM_006242885	Hyou1	hypoxia up-regulated 1	protein_coding	NP_001029200.1;NP_620222.2;XP_006242946.1;XP_006242947.1	-2.266148
192247	NM_001105754, XM_006246953, XM_006246954, XM_017596980,	Sez6	seizure related 6 homolog	protein_coding	NP_001099224.2;XP_006247015.1;XP_006247016.1;XP_017452469.1;XP_017452470.1;XP_017452471.1	2.084068

	XM_017596981, XM_017596982					
192280	NM_138912, XM_006253215, XM_006253216	Ppp1r3b	protein phosphatase 1, regulatory subunit 3B	protein_coding	NP_620267.2;XP_006253277.1;XP_006253278.1	2.534819
192362	NM_001100640, XM_008769603	Lamc2	laminin subunit gamma 2	protein_coding	NP_001094110.1;XP_008767825.1	-2.193524
246245	NM_144737, XM_008769604, XM_017598668	Fmo2	flavin containing monooxygenase 2	protein_coding	NP_653338.2;XP_008767826.1;XP_017454157.1	2.123909
266778	NM_001099498, XM_006232438, XM_008761031, XM_017590654, XM_017590655	Vom2r44	vomeronasal 2 receptor 44	protein_coding	NP_001092968.1;XP_006232500.1;XP_008759253.1;XP_017446143.1;XP_017446144.1	2.134672
286976	NM_173312, XM_006243366, XM_008766220, XM_017595498, XM_017595499	Gcnt3	glucosaminyl (N-acetyl) transferase 3, mucin type	protein_coding	NP_775434.1;XP_006243428.1;XP_008764442.1;XP_017450987.1;XP_017450988.1	2.261265
287101	NM_001105766, XM_006245868, XM_017597058, XM_017597059, XM_017597060	Pkmyt1	protein kinase, membrane associated tyrosine/threonine 1	protein_coding	NP_001099236.1;XP_006245930.1;XP_017452547.1;XP_017452548.1;XP_017452549.1	-2.120402
287422	NM_001034125, XM_006246613, XM_006246614	Per1	period circadian clock 1	protein_coding	NP_001029297.1;XP_006246675.1;XP_006246676.1	6.949080
288499	NM_001105911	Slc29a4	solute carrier family 29 member 4	protein_coding	NP_001099381.1	2.957060
288529	XM_221997	Gjc3	gap junction protein, gamma 3	protein_coding	XP_221997.5	2.607036

289486	NM_001191595	Fras1	Fraser extracellular matrix complex subunit 1	protein_coding	NP_001178524.1	6.174221
289533	NM_001135869	Ugt2a3	UDP glucuronosyltransferase 2 family, polypeptide A3	protein_coding	NP_001129341.1	2.135424
291005	NM_001077640	Gadd45g	growth arrest and DNA-damage-inducible, gamma	protein_coding	NP_001071108.1	-2.962179
291081	NM_001013886	Tubb2b	tubulin, beta 2B class IIb	protein_coding	NP_001013908.2	-2.137245
291352	NM_001170326	Gpr158	G protein-coupled receptor 158	protein_coding	NP_001163797.1	5.239956
291648	XM_017601096	Pcdhb11	protocadherin beta 11	protein_coding	XP_017456585.1	-2.317603
291654	NM_001114602	Pcdhb5	protocadherin beta 5	protein_coding	NP_001108074.1	-2.429461
291966	NM_001009639, XM_006255464	Tppp3	tubulin polymerization-promoting protein family member 3	protein_coding	NP_001009639.1;XP_006255526.1	-3.153862
294335	NM_001106381, XM_006256338	Susd2	sushi domain containing 2	protein_coding	NP_001099851.1;XP_006256400.1	2.531136
295462	XM_006233280, XM_017591345	Mcub	mitochondrial calcium uniporter dominant negative beta subunit, transcript variant X1	protein_coding	XP_006233342.1	-2.601026
296178	NM_001106514, XM_006235074, XM_008762163	Mcm8	minichromosome maintenance 8 homologous recombination repair factor	protein_coding	NP_001099984.1;XP_006235136.1;XP_008760385.1	2.955056
296952	XM_008762841, XM_017592967, XM_017592968, XM_017592969	Kcp	kielin/chordin-like protein, transcript variant X4	protein_coding	XP_008761063.1;XP_017448456.1;XP_017448457.1;XP_017448458.1	2.603864
297902	NM_001106637	Gem	GTP binding protein	protein_coding	NP_001100107.1	-2.250845

			overexpressed in skeletal muscle			
298504	NM_001106683, XM_006238792	Mfsd2a	major facilitator superfamily domain containing 2A	protein_coding	NP_001100153.1;XP_006238854.1	2.928048
299511	NM_199208	Rdh16	retinol dehydrogenase 16 (all-trans)	protein_coding	NP_954678.1	2.084362
299762	NM_001171177, XM_017594739	Tmtc2	transmembrane and tetratricopeptide repeat containing 2	protein_coding	NP_001164648.2;XP_017450228.1	-3.783624
301323	NM_001024762, XM_006244686, XM_006244689, XM_006244690, XM_006244691, XM_017596335, XM_017596336, XM_017596337	Prim2	primase, DNA, polypeptide 2	protein_coding	NP_001019933.1;XP_006244748.1;XP_006244751.1;XP_006244752.1;XP_006244753.1;XP_017451824.1;XP_017451825.1;XP_017451826.1	2.145502
302327	XM_002730265, XM_008773361, XM_017602345, XM_017602346, XM_017602347, XM_017602348	Zfp711	zinc finger protein 711, transcript variant X1	protein_coding	XP_002730311.1;XP_008771583.1;XP_017457834.1;XP_017457835.1;XP_017457836.1;XP_017457837.1	-2.074825
302424	NM_001305243, XM_006257111, XM_006257112, XM_008773301, XM_017601968, XM_017601969, XM_228582	Eda	ectodysplasin-A	protein_coding	NP_001292172.1;XP_006257173.1;XP_006257174.1;XP_008771523.1;XP_017457457.1;XP_017457458.1;XP_228582.6	2.076364
302970	NM_001106983	Prss32	protease, serine, 32	protein_coding	NP_001100453.1	4.070154

303021	XM_006246109, XM_017597615, XM_017597616	Sh3pxd2b	SH3 and PX domains 2B, transcript variant X3	protein_coding	XP_006246171.1;XP_017453104.1;XP_017453105.1	2.673666
303384	NM_001079888	Mmp28	matrix metallopeptidase 28	protein_coding	NP_001073357.1	-2.207905
303836	NM_001107084	Bcl6	B-cell CLL/lymphoma 6	protein_coding	NP_001100554.1	-4.773959
304445	XM_017598512, XM_222147	Glt1d1	glycosyltransferase 1 domain containing 1, transcript variant X2	protein_coding	XP_017454001.1;XP_222147.4	3.108555
305365	XM_223434	Slc25a52	solute carrier family 25, member 52	protein_coding	XP_223434.2	3.085422
305540	NM_198763	Slc1a4	solute carrier family 1 member 4	protein_coding	NP_942058.1	-2.292556
305842	NM_206815, XM_006251871, XM_006251872, XM_008770519, XM_008770520, XM_017599665, XM_017599666	Rnase6	ribonuclease, RNase A family, 6	protein_coding	NP_996538.1;XP_006251933.1;XP_006251934.1;XP_008768741.1;XP_008768742.1;XP_017455154.1;XP_017455155.1	-3.063368
306506	NM_001107315, XM_017600131, XM_017600132	Pragmin	pragma of Rnd2	protein_coding	NP_001100785.1;XP_017455620.1;XP_017455621.1	-2.240272
306810	NM_001107341, XM_006253709	Susd3	sushi domain containing 3	protein_coding	NP_001100811.1;XP_006253771.1	-2.966984
306934	NM_001014009, XM_006253929, XM_006253930, XM_008771637, XM_017600530, XM_017600531, XM_017600532,	Fam65b	family with sequence similarity 65, member B	protein_coding	NP_001014031.2;XP_006253991.1;XP_006253992.1;XP_008769859.1;XP_017456019.1;XP_017456020.1;XP_017456021.1;XP_017456022.1;XP_017456023.1;XP_017456024.1	9.635298

	XM_017600533, XM_017600534, XM_017600535					
306970	NM_001107354	Hist1h2an	histone cluster 1, H2an	protein_coding	NP_001100824.1	-2.226941
307035	NM_001100575, XM_017600560, XM_017600561, XM_017600562, XM_017600563, XM_017600564	Mpp7	membrane palmitoylated protein 7	protein_coding	NP_001094045.1;XP_017456049.1;XP_017456050.1;XP_017456051.1;XP_017456052.1;XP_017456053.1	2.416090
307126	NM_001107366	Mcm10	minichromosome maintenance 10 replication initiation factor	protein_coding	NP_001100836.1	3.425481
307253	XM_006254971, XM_006254972, XM_008772209, XM_008772210, XM_017601136, XM_017601137	Katnal2	katanin catalytic subunit A1 like 2, transcript variant X2	protein_coding	XP_006255033.1;XP_006255034.1;XP_008770431.1;XP_008770432.1;XP_017456625.1;XP_017456626.1	-2.018052
307305	XM_008772189, XM_008772190, XM_008772191	Prdm6	PR domain 6, transcript variant X3	protein_coding	XP_008770411.1;XP_008770412.1;XP_008770413.1	-2.606104
307412	XM_017601111, XM_225905	RGD1305184	similar to CDNA sequence BC023105, transcript variant X1	protein_coding	XP_017456600.1;XP_225905.4	-2.731622
307414	NM_001012353	MGC108823	similar to interferon-inducible GTPase	protein_coding	NP_001012353.1	-2.291792
307415	NM_001024884, XM_006254759	RGD1309362	similar to interferon-inducible GTPase	protein_coding	NP_001020055.1;XP_006254821.1	-3.771177
307916	NM_001107437	Jph3	junctophilin 3	protein_coding	NP_001100907.1	5.307473
308099	NM_001038615,	Fndc1	fibronectin type III	protein_coding	NP_001033704.1;XP_006227933.1	-2.192246

	XM_006227871		domain containing 1			
308843	NM_001009965, XM_006229759, XM_006229760, XM_006229761	Tsku	tsukushi, small leucine rich proteoglycan	protein_coding	NP_001009965.1;XP_006229821.1;XP_006229822.1;XP_006229823.1	6.615266
308937	NM_001012742, XM_006229974, XM_017589203	Wee1	WEE1 G2 checkpoint kinase	protein_coding	NP_001012760.1;XP_006230036.1;XP_017444692.1	2.857321
309122	NR_027324	H19	H19, imprinted maternally expressed transcript (non-protein coding)	lncRNA	.	-2.120305
309300	XM_017590330, XM_017590331, XM_017590332, XM_017590333, XM_017590334, XM_017590335, XM_017590336, XM_017590337	RGD1565057	similar to hypothetical protein FLJ32871, transcript variant X2	protein_coding	XP_017445819.1;XP_017445820.1;XP_017445821.1;XP_017445822.1;XP_017445823.1;XP_017445824.1;XP_017445825.1;XP_017445826.1	2.701612
310132	NM_001005384, XM_006232012	Osmr	oncostatin M receptor	protein_coding	NP_001005384.1;XP_006232074.1	-2.063355
310200	NM_001191565	Ankrd33b	ankyrin repeat domain 33B	protein_coding	NP_001178494.1	2.256389
310376	XM_006231982, XM_227081	Nim1k	NIM1 serine/threonine protein kinase, transcript variant X1	protein_coding	XP_006232044.1;XP_227081.3	-2.671884
310395	NM_138526	Noct	nocturnin	protein_coding	NP_612535.1	3.064208
310405	NM_001107675	Maml3	mastermind-like transcriptional coactivator 3	protein_coding	NP_001101145.2	2.200719
310508	NM_001013158, XM_017590867,	B3galnt1	beta-1,3-N-acetylgalactosaminyltransf	protein_coding	NP_001013176.1;XP_017446356.1;XP_017446357.1;XP_017446358.1;XP_0174463	-2.681124

	XM_017590868, XM_017590869, XM_017590870		erase 1 (globoside blood group)		59.1	
310537	NM_001107685	RGD1560010	RGD1560010	protein_coding	NP_001101155.1	-2.072145
310877	NM_001014044, XM_017590938	Tifa	TRAF-interacting protein with forkhead-associated domain	protein_coding	NP_001014066.1;XP_017446427.1	-2.173043
311029	"XM_006234174, XM_006234175, XM_006234176, XM_006234177,	Neb	nebulin, transcript variant X3	protein_coding	XP_006234236.1;XP_006234237.1;XP_006234238.1;XP_006234239.1;XP_006234240.1;XP_006234241.1;XP_006234242.1;XP_006234243.1;XP_006234244.1;XP_006234245.1;XP_006234247.1;XP_006234248.1;XP_006234250.1;XP_006234252.1;XP_006234253.1;XP_006234254.1;XP_006234255.1;XP_006234256.1;XP_008760037.1;XP_008760038.1;XP_008760039.1;XP_008760040.1;XP_008760041.1;XP_008760042.1;XP_008760043.1;XP_017447634.1;XP_017447635.1;XP_017447636.1;XP_017447637.1	-4.004673
311336	XM_006234178,XM_006234179,XM_006234180,XM_006234181,XM_006234182,XM_006234183,XM_006234185,XM_006234186,XM_006234188,XM_006234190,XM_006234191,XM_006234192,XM_006234193,XM_006234194,XM_008761815,XM_008761816,XM_008761817,XM_008761818,	Nusap1	nucleolar and spindle associated protein 1	protein_coding	NP_001101232.1;XP_006234830.1;XP_006234831.1;XP_006234832.1;XP_008760323.1;XP_008760324.1	-2.606863

	XM_008761819,XM_008761820,XM_008761821,XM_017592145,XM_017592146,XM_017592147,XM_017592148"					
311642	NM_001107762,XM_006234768,XM_006234769,XM_006234770,XM_008762101,XM_008762102	Sulf2	sulfatase 2	protein_coding	NP_001030099.1;XP_006235658.1	2.196252
311901	NM_001034927,XM_006235596	Lhx6	LIM homeobox 6	protein_coding	NP_001101307.1;XP_006234128.1;XP_008759975.1	2.286536
312213	NM_001107837,XM_006234066,XM_008761753	Plxna4	plexin A4	protein_coding	XP_017448459.1	2.030152
312246	XM_017592970	RGD1306271	similar to KIAA1549 protein, transcript variant X6	protein_coding	XP_006236400.1;XP_006236403.1;XP_008761069.1;XP_017448461.1;XP_017448462.1;XP_017448463.1;XP_231616.5	2.044846
312495	XM_006236338,XM_006236341,XM_008762847,XM_017592972,XM_017592973,XM_017592974,XM_231616	Cyp26b1	cytochrome P450, family 26, subfamily b, polypeptide 1	protein_coding	NP_851601.1	-2.200175
313022	NM_181087	Map3k6	mitogen-activated protein kinase kinase kinase 6	protein_coding	NP_001101379.1;XP_006239081.1;XP_006239082.1;XP_006239083.1;XP_006239084.1;XP_008762359.1;XP_017448820.1;XP_017448821.1	2.493880
313722	NM_001107909,XM_006239019,XM_006239020,XM_006239021,XM_006239022,XM_008764137,XM_017593331,XM_017593332	Spsb1	splA/ryanodine receptor domain and SOCS box containing 1	protein_coding	NP_001101464.1;XP_006239524.1;XP_008762479.1;XP_008762480.1;XP_008762481.1;XP_017448919.1	2.258511
313993	NM_001107994,XM_006	Grhl1	grainyhead-like	protein_coding	XP_234006.5	-2.223198

	239462,XM_008764257,XM_008764258,XM_008764259,XM_017593430		transcription factor 1, transcript variant X1			
314322	XM_234006	Fos	FBJ osteosarcoma oncogene	protein_coding	NP_071533.1	-3.820698
314616	NM_022197	Arid3a	AT-rich interaction domain 3A	protein_coding	NP_001101536.2;XP_006240964.1;XP_006240966.1;XP_006240967.1;XP_008763391.1;XP_017450301.1	-2.579926
315711	NM_001108066,XM_006240902,XM_006240904,XM_006240905,XM_008765169,XM_017594812	Sema7a	semaphorin 7A, GPI membrane anchor	protein_coding	NP_001101623.1;XP_017451142.1	-2.047154
315714	NM_001108153,XM_017595653	Loxl1	lysyl oxidase-like 1	protein_coding	NP_001012125.1	-2.027711
316304	NM_001012125	Lgsn	lengsin, lens protein with glutamine synthetase domain	protein_coding	NP_852048.1;XP_017451925.1;XP_017451926.1	-2.872359
316351	NM_181383,XM_017596436,XM_017596437	Npas2	neuronal PAS domain protein 2	protein_coding	NP_001101684.2;XP_008765253.1;XP_008765254.1;XP_008765255.1	-4.581819
316444	NM_001108214,XM_008767031,XM_008767032,XM_008767033	Mdh1b	malate dehydrogenase 1B	protein_coding	NP_001101691.1;XP_017451958.1;XP_017451959.1;XP_017451960.1;XP_017451961.1;XP_017451962.1	-2.159311
316539	NM_001108221,XM_017596469,XM_017596470,XM_017596471,XM_017596472,XM_017596473	Epha4	Eph receptor A4	protein_coding	NP_001155883.1;XP_008765471.1	-6.036169
316600	NM_001162411,XM_008767249	Ugt1a9-ps	UDP glycosyltransferase 1 family, polypeptide A9, pseudogene	.	.	-2.051591
316736	gene22704	Emilin2	elastin microfibril interfacer 2, transcript variant X1	protein_coding	XP_017452370.1;XP_017452371.1	-2.125815

353227	XM_017596881,XM_017596882	Zbtb16	zinc finger and BTB domain containing 16	protein_coding	NP_001013199.1;XP_017451194.1;XP_017451195.1;XP_017451196.1	6.557909
360323	NM_001013181,XM_017595705,XM_017595706,XM_017595707	RT1-N2	RT1 class Ib, locus N2	protein_coding	NP_001008854.1;XP_006256000.1;XP_017457159.1;XP_017457160.1;XP_017457161.1	2.160349
360711	NM_001008854,XM_006255938,XM_017601670,XM_017601671,XM_017601672	RGD1310335	similar to RIKEN cDNA C330027C09	protein_coding	XP_340983.3	-2.958364
360793	XM_340982	Rhbdd2	rhomboid domain containing 2	protein_coding	NP_001178756.1;XP_006249244.1	2.512228
360896	NM_001191827,XM_006249182	Esrrg	estrogen-related receptor gamma	protein_coding	NP_976081.1;XP_006250505.1;XP_017454353.1;XP_017454354.1;XP_017454355.1;XP_017454356.1;XP_017454357.1;XP_017454358.1;XP_017454359.1	2.061769
360959	NM_203336,XM_006250443,XM_017598864,XM_017598865,XM_017598866,XM_017598867,XM_017598868,XM_017598869,XM_017598870	Htra3	HtrA serine peptidase 3	protein_coding	NP_001257956.1;XP_017454804.1	-2.078586
361034	NM_001271027,XM_017599315	Arhgef40	Rho guanine nucleotide exchange factor 40	protein_coding	NP_001258242.1	2.132462
361084	NM_001271313,XR_001841260	Lmo7	LIM domain 7	protein_coding	NP_001001515.1;XP_006252459.1;XP_006252461.1;XP_006252462.1;XP_006252463.1;XP_017455238.1;XP_017455239.1;XP_017455240.1;XP_017455241.1;XP_017455242.1;XP_017455243.1	2.399935
361187	NM_001001515,XM_006252397,XM_006252399,XM_006252400,XM_006252401,XM_017599749,	Asb5	ankyrin repeat and SOCS box-containing 5	protein_coding	NP_001037712.1;XP_006253162.1;XP_008769469.1;XP_008769470.1;XP_017455679.1;XP_017455680.1	2.043631

	XM_017599750,XM_017599751,XM_017599752,XM_017599753,XM_017599754					
361453	NM_001044247,XM_006253100,XM_008771247,XM_008771248,XM_017600190,XM_017600191	Adat2	adenosine deaminase, tRNA-specific 2	protein_coding	NP_001108500.1	-2.262144
361795	NM_001115028	Ltb	lymphotoxin beta	protein_coding	NP_997672.1	-2.758288
362042	NM_212507	Gimd1	GIMAP family P-loop NTPase domain containing 1	protein_coding	NP_001107254.2;XP_006233379.1;XP_008759738.1;XP_017446470.1;XP_017446471.1	-3.094394
362131	NM_001113782,XM_006233317,XM_008761516,XM_017590981,XM_017590982	Max-ps1	Max protein, pseudogene 1	.	.	-2.702321
362336	gene8372	Fam180a	family with sequence similarity 180, member A	protein_coding	NP_001102091.1	2.032149
362360	NM_001108621	Osbpl3	oxysterol binding protein-like 3, transcript variant X9	protein_coding	XP_006236577.1;XP_006236578.1;XP_006236579.1;XP_006236580.1;XP_006236581.1;XP_008761156.1;XP_008761157.1;XP_017448483.1;XP_342684.5	3.132547
362539	XM_006236515,XM_006236516,XM_006236517,XM_006236518,XM_006236519,XM_008762934,XM_008762935,XM_017592994,XM_342683	Adamtsl1	ADAMTS-like 1, transcript variant X1	protein_coding	XP_017449410.1	-2.208798
362558	XM_017593921	Lrp8	LDL receptor related protein 8, transcript variant X17	protein_coding	XP_003750040.1;XP_003750041.1;XP_006238629.1;XP_006238632.1;XP_006238633.1;XP_006238636.1;XP_008762159.1;XP_008762160.1;XP_008762161.1;XP_008	-2.863375

					762163.1;XP_008762164.1;XP_008762165.1;XP_008762166.1;XP_008762167.1;XP_008762168.1;XP_008762170.1;XP_342878.5	
362675	XM_003749992,XM_003749993,XM_006238567,XM_006238570,XM_006238571,XM_006238574,XM_008763937,XM_008763938,XM_008763939,XM_008763941,XM_008763942,XM_008763943,XM_008763944,XM_008763945,XM_008763946,XM_008763948,XM_342877	Tp73	tumor protein p73	protein_coding	NP_001102166.1	4.157447
362790	NM_001108696	Inf2	inverted formin, FH2 and WH2 domain containing, transcript variant X1	protein_coding	XP_017450035.1	2.087506
362795	XM_017594546	LOC362795	.	.	.	-2.232863
362972	gene16936,id317589	Pnpla3	patatin-like phospholipase domain containing 3	protein_coding	NP_001269253.1;XP_017450484.1	-4.617474
362978	NM_001282324,XM_017594995	Creld2	cysteine-rich with EGF-like domains 2	protein_coding	NP_001032285.1;XP_006242278.1	-3.608146
363588	NM_001037208,XM_006242216	RGD1559512	.	.	.	4.269269
363933	gene23674	LOC363933	.	.	.	-2.514649
364983	gene26918	Rnf150	ring finger protein 150	protein_coding	NP_001178022.1	2.512995
365748	NM_001191093	Bhlhe22	basic helix-loop-helix family, member e22	protein_coding	NP_001102410.1	-2.091294
365871	NM_001108940	Ciart	circadian associated	protein_coding	NP_001128065.1	4.421580

			repressor of transcription			
365960	NM_001134593	Gipc2	GIPC PDZ domain containing family, member 2	protein_coding	NP_001032287.1;XP_008759774.1	2.385371
366275	NM_001037210,XM_008761552	Ptk6	protein tyrosine kinase 6	protein_coding	NP_001102438.1	-2.608810
366962	NM_001108968	Grap2	GRB2-related adaptor protein 2	protein_coding	NP_001030116.1	-2.206746
414779	NM_001034944	RT1-CE2	RT1 class I, locus CE2	protein_coding	NP_001008840.1	-2.036888
414793	NM_001008840	RT1-CE3	RT1 class I, locus CE3	protein_coding	NP_001008841.1;XP_017457219.1;XP_017457220.1;XP_017457221.1;XP_017457222.1	-2.456342
415059	NM_001008841,XM_017601730,XM_017601731,XM_017601732,XM_017601733	Rpl41-ps1	ribosomal protein L41, pseudogene 1	.	.	-4.416809
445442	gene36335	Thbs1	thrombospondin 1	protein_coding	NP_001013080.1	-3.555069
497619	NM_001013062	Fut10	fucosyltransferase 10	protein_coding	NP_001012360.1;XP_006253298.1;XP_017455693.1;XP_017455694.1;XP_017455695.1	2.129617
498072	NM_001012360,XM_006253236,XM_017600204,XM_017600205,XM_017600206	Hmgb2l1	high mobility group box 2-like 1	protein_coding	XP_573272.3	-2.111241
498287	XM_573272	Slamf6	SLAM family member 6	protein_coding	NP_001178861.1;XP_006250363.1	-3.532164
498351	NM_001191932,XM_006250301,XR_595551	Srp72	signal recognition particle 72	protein_coding	NP_001164072.1	-3.832950
498736	NM_001170601	Tubb2a	tubulin, beta 2A class IIa	protein_coding	NP_001102589.1	-2.252835
498848	NM_001109119	RGD1560482	similar to protocadherin gamma B1	protein_coding	XP_008770294.1	-4.272622
499171	XM_008772072	Klf13	Kruppel-like factor 13	protein_coding	NP_001102617.1	2.829806
499723	NM_001109147	Col24a1	collagen, type XXIV, alpha	protein_coding	XP_017446891.1	2.617577

			1, transcript variant X1			
500175	XM_017591402	LOC500175	.	.	.	-3.031205
500679	gene8236,id146931	Rad51b	RAD51 paralog B, transcript variant X2	protein_coding	XP_017449963.1;XP_017449964.1;XP_017449965.1	-2.915016
501033	XM_017594474,XM_017594475,XM_017594476	LOC501033	similar to UPF0258 protein KIAA1024, transcript variant X3	protein_coding	XP_001055454.1;XP_017451604.1;XP_017451605.1	-2.021346
501582	XM_001055454,XM_017596115,XM_017596116,XR_001839494	Gspt2	G1 to S phase transition 2	protein_coding	NP_001102789.1	3.424536
502814	NM_001109319	RGD1564381	.	.	.	-2.669980
502844	gene8209,id146851	RGD1564861	.	.	.	-2.340949
553106	gene11834,id212551	Ncald	neurocalcin delta	protein_coding	NP_001019542.1;XP_008763678.1;XP_017450554.1;XP_017450555.1	-2.660033
574531	NM_001024371,XM_008765456,XM_017595065,XM_017595066	Lair1	leukocyte-associated immunoglobulin-like receptor 1	protein_coding	NP_001025099.1;XP_008757196.1;XP_017445109.1;XP_017445110.1;XP_017445111.1;XP_017445112.1;XP_017445113.1	3.613771
641521	NM_001029928,XM_008758974,XM_017589620,XM_017589621,XM_017589622,XM_017589623,XM_017589624	Lrrc4	leucine rich repeat containing 4	protein_coding	NP_001032413.1	2.105588
641603	NM_001037336	Slc41a3	solute carrier family 41, member 3	protein_coding	NP_001032569.1;XP_006236933.1;XP_006236934.1;XP_006236935.1;XP_006236936.1;XP_006236937.1;XP_006236939.1;XP_006236940.1	7.331435
679975	NM_001037492,XM_006236871,XM_006236872,XM_006236873,XM_006236874,XM_006236875,XM_006236877,XM_006236878	Gpr183	G protein-coupled receptor 183	protein_coding	NP_001102856.1	-2.607192

680110	NM_001109386	Rprm	reprimo, TP53 dependent G2 arrest mediator candidate	protein_coding	NP_001037741.1	-4.506902
680262	NM_001044276	Hyls1	HYLS1, centriolar and ciliogenesis associated, transcript variant X2	protein_coding	XP_002729970.1;XP_002729971.1	-2.385060
680329	XM_002729924,XM_002729925	LOC680329	immunoglobulin lambda-like polypeptide 5-like, transcript variant X3	protein_coding	XP_017453680.1;XP_017453681.1;XP_017453682.1;XP_017453683.1;XP_017453684.1;XP_017453685.1;XP_017453686.1;XP_017453687.1;XP_017453688.1	-2.542842
680409	XM_017598191,XM_017598192,XM_017598193,XM_017598194,XM_017598195,XM_017598196,XM_017598197,XM_017598198,XM_017598199	Prodh1	proline dehydrogenase (oxidase) 1	protein_coding	NP_001129250.1;XP_006248704.3;XP_006248705.1;XP_008767125.1	2.191482
680702	NM_001135778,XM_006248642,XM_006248643,XM_008768903,XR_001840423	Ripply1	ripply transcriptional repressor 1	protein_coding	XP_008771721.2	-2.117462
684050	XM_008773499	PCOLCE2	procollagen C-endopeptidase enhancer 2	protein_coding	NP_001121112.1	-2.939269
684762	NM_001127640	LOC684762	similar to CG31613-PA, transcript variant X1	protein_coding	XP_008769899.1	-2.031535
685448	XM_008771677	Pcp4l1	Purkinje cell protein 4-like 1	protein_coding	NP_001119565.1;XP_017454425.1	2.149122
685879	NM_001126093,XM_017598936	Jazf1	JAZF zinc finger 1, transcript variant X1	protein_coding	XP_001065610.1;XP_006236597.1;XP_006236598.1;XP_008761175.1;XP_017448489.1;XP_017448490.1	-2.201270
687064	XM_001065610,XM_006236535,XM_006236536,	Col25a1	collagen, type XXV, alpha 1, transcript variant X1	protein_coding	XP_017446835.1	2.135063

	XM_008762953,XM_017593000,XM_017593001					
687071	XM_017591346	Etnppl	ethanolamine-phosphate phospho-lyase, transcript variant X1	protein_coding	XP_003749444.1	2.545283
687508	XM_003749396	LOC687508	similar to Cytochrome c oxidase polypeptide VIIa-heart, mitochondrial precursor (Cytochrome c oxidase subunit VIIa-H) (COX VIIa-M), transcript variant X2	protein_coding	XP_003748883.1;XP_017445539.1;XP_017445540.1;XP_017445541.1;XP_017445542.1;XP_017445543.1;XP_017445544.1;XP_017445545.1	2.459563
687696	XM_003748835,XM_017590050,XM_017590051,XM_017590052,XM_017590053,XM_017590054,XM_017590055,XM_017590056,XR_001835721	Stambpl1	STAM binding protein-like 1	protein_coding	NP_001108520.1;XP_006231358.1;XP_006231359.1;XP_006231361.1;XP_008758568.1;XP_008758571.1;XP_017445215.1	-2.703405
689092	NM_001115048,XM_006231296,XM_006231297,XM_006231299,XM_008760346,XM_008760349,XM_017589726	Gcnt6	glucosaminyl (N-acetyl) transferase 6	protein_coding	XP_008769800.2	2.808835
689257	XM_008771578	Adgrd1	adhesion G protein-coupled receptor D1, transcript variant X2	protein_coding	XP_001070157.3;XP_017454000.1	2.852622
689906	XM_001070157,XM_017598511	LOC689906	.	.	.	-2.729439
690315	gene31257	Derl3	derlin 3	protein_coding	NP_001103047.2;XP_006256387.1;XP_006256388.1;XP_008771136.1;XP_008771137.1;XP_008771138.1;XP_008771139.1;XP_008771140.1;XP_008771142.1;XP_008	-2.232836

					771144.1;XP_008771145.1;XP_008771146.1;XP_017457265.1	
690919	NM_001109577,XM_006256325,XM_006256326,XM_008772914,XM_008772915,XM_008772916,XM_008772917,XM_008772918,XM_008772920,XM_008772922,XM_008772923,XM_008772924,XM_017601776	Nrg4	neuregulin 4	protein_coding	NP_001178038.1;XP_017451408.1	16.194004
691418	NM_001191109,XM_017595919	LOC691418	hypothetical protein LOC691418	protein_coding	XP_001068693.1	7.350779
691770	XM_001068693	Ankrd63	ankyrin repeat domain 63	protein_coding	XP_006234855.1	-2.613963
691828	XM_006234793	LOC691828	.	.	.	-2.892551
727679	gene17011,id317846	Pdxp	pyridoxal phosphatase	protein_coding	NP_001129291.1	3.179861
100301568	NM_001135819	Abo	ABO blood group (transferase A, alpha 1-3-N-acetylgalactosaminyltransferase; transferase B, alpha 1-3-galactosyltransferase)	protein_coding	NP_001153735.1	-2.705854
100360186	NM_001160263	LOC100360186	.	.	.	-2.437685
100361087	gene20056	LOC100361087	hypothetical LOC100361087	protein_coding	NP_001177388.1	-2.000074
100361238	NM_001190459	Rasgef1b	RasGEF domain family, member 1B, transcript variant X3	protein_coding	XP_002728129.2;XP_017454916.1;XP_017454917.1;XP_017454918.1	2.004514
100361460	XM_002728083,XM_017599427,XM_017599428,XM_017599429	Fam196b	family with sequence similarity 196, member B	protein_coding	XP_008765845.1	2.374405
100362624	XM_008767623	LOC100362624	.	.	.	-3.044311

100910821	gene5406	LOC100910821	uncharacterized LOC100910821	lncRNA	.	-2.886172
100910934	XR_592911	LOC100910934	interferon-inducible GTPase 1-like	protein_coding	XP_003751840.2	-3.813806
100910979	XM_003751792	LOC100910979	interferon-inducible GTPase 1-like, transcript variant X1	protein_coding	XP_006254820.1	-2.295331
100911029	XM_006254758	LOC100911029	nudC domain-containing protein 3-like, transcript variant X1	lncRNA	.	6.014997
100911038	XR_001841167,XR_359658	LOC100911038	uncharacterized LOC100911038, transcript variant X1	lncRNA	.	2.389723
100911657	XR_001838986	LOC100911657	.	.	.	-2.005073
100912124	gene20057	LOC100912124	uncharacterized LOC100912124, transcript variant X1	lncRNA	.	-2.428328
100912801	XR_001841748,XR_596586	LOC100912801	.	.	.	2.157110
102546403	gene11601	LOC102546403	uncharacterized LOC102546403, transcript variant X1	lncRNA	.	-2.409215
102546824	XR_594576,XR_594577	LOC102546824	uncharacterized LOC102546824	lncRNA	.	-2.705167
102546876	XR_353305	LOC102546876	.	.	.	-3.754817
102547038	gene16932,id317570	LOC102547038	uncharacterized LOC102547038, transcript variant X1	lncRNA	.	2.257214
102547431	XR_591088,XR_591089	LOC102547431	uncharacterized LOC102547431	protein_coding	XP_017449176.1	2.517534
102547502	XM_017593687	LOC102547502	uncharacterized LOC102547502, transcript	lncRNA	.	2.450885

			variant X1			
102547508	XR_001838114,XR_001838115	LOC102547508	uncharacterized LOC102547508, transcript variant X2	lncRNA	.	3.200248
102548187	XR_355579,XR_593482	LOC102548187	.	.	.	-3.195690
102548231	gene15384	LOC102548231	zinc finger protein 850-like, transcript variant X4	protein_coding	XP_006228981.1;XP_006228982.1;XP_017445562.1;XP_017445563.1;XP_017445564.1	-2.366019
102548289	XM_006228919,XM_006228920,XM_017590073,XM_017590074,XM_017590075	LOC102548289	uncharacterized LOC102548289	lncRNA	.	3.921281
102548588	XR_001839782	LOC102548588	.	.	.	-2.124258
102548801	gene33483	LOC102548801	uncharacterized LOC102548801	lncRNA	.	-2.935827
102548978	XR_359861	LOC102548978	uncharacterized LOC102548978, transcript variant X1	protein_coding	XP_006255270.1	6.629804
102549109	XM_006255208,XR_597143	LOC102549109	uncharacterized LOC102549109, transcript variant X4	lncRNA	.	-2.133746
102549302	XR_597017,XR_597018,XR_597019,XR_597020	LOC102549302	.	.	.	3.122662
102549496	gene28202	LOC102549496	uncharacterized LOC102549496	lncRNA	.	-2.514179
102549537	XR_353949	LOC102549537	uncharacterized LOC102549537, transcript variant X3	lncRNA	.	-3.524991
102549616	XR_001839299,XR_001839300,XR_001839301	LOC102549616	uncharacterized LOC102549616	lncRNA	.	-3.224896
102549760	XR_590323	LOC102549760	uncharacterized LOC102549760, transcript	lncRNA	.	-4.398352

			variant X1			
102549876	XR_361987,XR_597317	LOC102549876	uncharacterized LOC102549876	lncRNA	.	-2.547783
102549932	XR_353952	LOC102549932	uncharacterized LOC102549932	lncRNA	.	-2.744362
102550012	XR_358411	LOC102550012	uncharacterized LOC102550012	lncRNA	.	2.988933
102550357	XR_351842	LOC102550357	uncharacterized LOC102550357	lncRNA	.	2.267852
102550525	XR_355973	LOC102550525	uncharacterized LOC102550525	lncRNA	.	2.189391
102550543	XR_358904	LOC102550543	uncharacterized LOC102550543	lncRNA	.	2.626144
102551861	XR_352181	LOC102551861	.	.	.	3.089970
102552123	gene31897	LOC102552123	uncharacterized LOC102552123, transcript variant X1	lncRNA	.	2.191146
102552469	XR_001842111,XR_597009	LOC102552469	uncharacterized LOC102552469, transcript variant X1	lncRNA	.	-3.000157
102553136	XR_596584,XR_596585	LOC102553136	uncharacterized LOC102553136	lncRNA	.	-3.975420
102553680	XR_354845	LOC102553680	uncharacterized LOC102553680, transcript variant X4	lncRNA	.	-2.346211
102554096	XR_001840529,XR_001840530,XR_001840531,XR_358328	LOC102554096	guanylate-binding protein 6-like, transcript variant X1	protein_coding	XP_006250672.1	-2.912051
102554269	XM_006250610	LOC102554269	uncharacterized LOC102554269, transcript variant X3	lncRNA	.	-4.184179
102554576	XR_001838802,XR_0018	LOC102554576	protein argonaute-4-like	protein_coding	XP_006227744.1	2.419294

	38803,XR_001838804,XR_001838805					
102555308	XM_006227682	LOC102555308	uncharacterized LOC102555308, transcript variant X3	protein_coding	XP_008768103.1;XP_008768104.1	2.365379
102555392	XM_008769881,XM_008769882,XR_595596,XR_595597	LOC102555392	interferon-inducible GTPase 1-like	protein_coding	XP_006254819.1	-2.221164
102555634	XM_006254757	LOC102555634	interferon-inducible GTPase 1-like	protein_coding	XP_006246477.2	-2.030752
102555850	XM_006246415	LOC102555850	.	.	.	-2.471361
102555924	gene8660	LOC102555924	uncharacterized LOC102555924, transcript variant X2	lncRNA	.	2.050530
102556085	XR_001837893,XR_001837894	LOC102556085	sodium/nucleoside cotransporter 2-like	protein_coding	XP_006234952.1	-2.831990
102556129	XM_006234890	LOC102556129	uncharacterized LOC102556129, transcript variant X1	protein_coding	XP_017455424.1	-2.342955
102556259	XM_017599935	LOC102556259	uncharacterized LOC102556259, transcript variant X1	misc_RNA	.	-2.720034
102556346	XR_001838773,XR_001838774,XR_001838775,XR_001838776	LOC102556346	angiotensin-converting enzyme-like	protein_coding	XP_006247705.1	2.569045
103690063	XM_006247643	LOC103690063	ATP-binding cassette sub-family G member 3-like	protein_coding	XP_008768187.1	-2.022343
103690146	XM_008769965	LOC103690146	.	.	.	-2.552538
103690360	gene16933,id317577	LOC103690360	.	.	.	-2.636761
103691422	gene11832,id212547	LOC103691422	uncharacterized LOC103691422, transcript variant X3	lncRNA	.	-2.070031

103691652	XR_001836536,XR_001836537,XR_590822	LOC103691652	uncharacterized LOC103691652, transcript variant X8	lncRNA	.	2.304416
103691897	"XR_001836834,XR_001836835,XR_001836836,XR_001836837,	LOC103691897	uncharacterized LOC103691897	lncRNA	.	-2.720390
103692723	XR_001836838,XR_001836839,XR_001836840,XR_001836841,XR_591243"	LOC103692723	.	.	.	-3.320419
103692951	XR_591692	LOC103692951	uncharacterized LOC103692951, transcript variant X1	lncRNA	.	2.059435
103693392	gene16934,id317582	LOC103693392	uncharacterized LOC103693392	lncRNA	.	2.221809
103693549	XR_593661	LOC103693549	uncharacterized LOC103693549	lncRNA	.	-6.181204
103694158	XR_594817	LOC103694158	uncharacterized LOC103694158	protein_coding	XP_008770182.1	-4.421682
103694226	XR_596118	LOC103694226	interferon-inducible GTPase 1-like	protein_coding	XP_008770418.1	-2.324001
103694227	XM_008771960	LOC103694227	uncharacterized LOC103694227	lncRNA	.	-2.388483
103694381	XM_008772196	LOC103694381	lymphotoxin-beta	protein_coding	XP_008770998.1	-2.780262
108348155	XR_597021	LOC108348155	paired immunoglobulin-like type 2 receptor beta-2, transcript variant X2	protein_coding	XP_008767322.1;XP_017454102.1;XP_017454103.1	2.956391
108348176	XM_008772776	LOC108348176	tubulin beta-2A chain pseudogene	misc_RNA	.	-2.383171
108348404	XM_008769100,XM_017598613,XM_017598614	LOC108348404	.	.	.	3.758902
108348437	XR_001841892	LOC108348437	.	.	.	-2.226394
108349443	gene32470	LOC108349443	uncharacterized	lncRNA	.	-2.323364

			LOC108349443			
108349722	gene32051	LOC108349722	sialic acid-binding Ig-like lectin 9	protein_coding	XP_017445897.1	-2.295282
108349760	XR_001835565	LOC108349760	dentin sialophosphoprotein-like	protein_coding	XP_017445934.1	-4.849067
108349911	XM_017590408	LOC108349911	.	.	.	2.089033
108350175	XM_017590445	LOC108350175	.	.	.	-2.173173
108351299	gene5010	LOC108351299	uncharacterized LOC108351299, transcript variant X2	lncRNA	.	-2.089666
108351309	gene5692	LOC108351309	.	.	.	-4.137426
108351310	XR_001838575,XR_001838576	LOC108351310	.	.	.	-3.579064
108351431	gene16972,id317729	LOC108351431	uncharacterized LOC108351431	protein_coding	XP_017450690.1	-2.079037
108351448	gene16970,id317723	LOC108351448	uncharacterized LOC108351448, transcript variant X3	lncRNA	.	-2.601806
108351964	XM_017595201	LOC108351964	uncharacterized LOC108351964, transcript variant X2	lncRNA	.	-6.240096
108352728	XR_001838833,XR_001838834,XR_001838835	LOC108352728	uncharacterized LOC108352728, transcript variant X2	lncRNA	.	2.137902
108353136	XR_001839869,XR_001839870	LOC108353136	uncharacterized LOC108353136	lncRNA	.	2.893103
108353140	XR_001841095,XR_001841096	LOC108353140	enhancer of filamentation 1-like	protein_coding	XP_017456246.1	-2.194682
